# The effectiveness of third wave cognitive behavioural therapies for children and adolescents: A systematic review and meta‐analysis

**DOI:** 10.1111/bjc.12404

**Published:** 2022-11-28

**Authors:** Amorette M. Perkins, Richard Meiser‐Stedman, Samuel W. Spaul, Gemma Bowers, Abigail G. Perkins, Laura Pass

**Affiliations:** ^1^ Department of Clinical Psychology and Psychological Therapies University of East Anglia Norfolk UK; ^2^ Norfolk and Suffolk NHS Foundation Trust Mary Chapman House Norfolk UK

**Keywords:** adolescent mental health, child mental health, meta‐analysis, third wave cognitive behavioural therapy

## Abstract

**Objectives:**

Third wave cognitive behavioural therapies are increasingly used with children and adolescents. This meta‐analysis aimed to determine the effectiveness of four third‐wave interventions (acceptance and commitment therapy, compassion focused therapy, mindfulness‐based cognitive therapy, and metacognitive therapy) for youth.

**Methods:**

Four electronic databases were used to identify randomized controlled trials, which tested effects related to health, well‐being and functioning. Sensitivity analyses considering study quality were conducted and moderators were explored.

**Results:**

The results based on 50 RCTs meeting inclusion criteria indicated emotional symptoms/internalizing problems (*g* = −.68, 95% CI −.98 to −.37, *k* = 43, *N* = 3265), behavioural difficulties/externalizing problems (*g* = −.62, 95% CI −1.01 to −.22, *k* = 23, *N* = 1659), interference from difficulties (*g* = −.46, 95% CI −.87 to −.05, *k* = 21, *N* = 1786), third wave processes (*g* = .39, 95% CI .17 to .62, *k* = 22, *N* = 1900), wellbeing/flourishing (*g* = .76, 95% CI .35 to 1.17, *k* = 21, *N* = 1303) and physical health/pain (*g* = .72, 95% CI .01 to 1.44, *k* = 9, *N* = 1171) yielded significant effects. Effect for quality of life (*g* = .62, 95% CI −.08 to 1.31, *k* = 12, *N* = 1271) was non‐significant. When analysing only those studies rated moderate‐high quality, third wave interventions yielded significant superiority effects compared to controls for emotional symptoms/internalizing problems (*g* = −.55, 95% CI −.82 to −.27, *k* = 28, *N* = 2110), interference from difficulties (*g* = −.48, 95% CI −.90 to −.05, *k* = 21, *N* = 1605), third wave processes (*g* = .27, 95% CI .11 to .43, *k* = 18, *N* = 1692), well‐being/flourishing (*g* = .50, 95% CI .18 to .81, *k* = 16, *N* = 1063), and quality of life (*g* = .32, 95% CI .04 to .60, *k* = 10, *N* = 1212). Behavioural difficulties/externalizing problems (*g* = −.38, 95% CI −.86 to .10, *k* = 15, *N* = 1351) and physical health/pain (*g* = .52, 95% CI −.14 to 1.17, *k* = 8, *N* = 1139) ceased to be significant. Widespread heterogeneity raised concerns about generalizability and follow‐up data was relatively sparse.

**Conclusions:**

This meta‐analysis finds promising results for use of third wave CBT with youth, though the review has limitations.

**Practitioner points**

- This review considers the effectiveness of four types of third wave CBT as a transdiagnostic approach for children and adolescents.
- When low quality studies were excluded, results indicated promising effects for a variety of outcomes related to psychological symptoms, well‐being and functioning.
- The evidence in this review was limited by considerable heterogeneity, with moderation and subgroup analyses offering limited explanation and restricted by current availability of research.
- Significant effects for emotional symptoms/internalizing problems were maintained at follow‐up, however analyses suggested no long‐term benefits for other outcomes. There were nonetheless limitations to follow‐up analyses that need to be addressed.

## INTRODUCTION

The term “third wave cognitive behavioural therapy (CBT)” refers to a group of psychological interventions that have been developed out of the existing cognitive behavioural interventions over recent decades (Brown et al., 2011). Many third wave methods are argued to offer a transdiagnostic approach, targeting common psychological processes relevant across the continuum from ill‐health to flourishing, rather than specific models of disorder or disease (Hayes & Hofmann, 2017). Interventions that could be universally applied to improve youth mental health and well‐being, offering continuity across levels of public health, are of particular interest given rising mental health issues amongst children and adolescents (Public Health England [PHE], 2019; UK Department of Health [DoH] & Department for Education [DfE], 2017).

Third wave therapies have thus gained increasing attention for use in younger populations, being implemented across presentations related to physical health, mental health, and substance use, as well as within neurodiverse groups and those at social disadvantage including children in care (e.g. Kallesoe et al., 2021; Kashefinishabouri et al., 2021; Makki et al., 2018; Pahnke et al., 2014; Thurstone et al., 2017; Wicksell et al., 2009). Third wave interventions have been delivered in clinics, schools, communities and via online platforms across the spectrum from treatment and prevention of ill‐health to the promotion of wellbeing and flourishing (e.g. Burckhardt et al., 2016; Syeda & Andrews, 2021; Twohig et al., 2021; White et al., 2022). Despite its widespread use, the effectiveness of third wave CBT as a transdiagnostic approach remains unclear, with no known meta‐analysis to synthesize existing data across child and youth populations to estimate effectiveness for a variety of presentations and outcomes.

Several therapies have been classified under the umbrella term of “third wave” approaches (O'Brien et al., 2008). The current article focused on four of the most recently developed third wave therapies; each using transdiagnostic cognitive and behavioural techniques with relevance along the continuum of ill‐health to flourishing. These were acceptance and commitment therapy (ACT; Hayes et al., 1999), compassion focused therapy (CFT; Gilbert, 2010), mindfulness‐based cognitive therapy (MBCT; Segal et al., 2002) and metacognitive therapy (MCT; Wells, 2009). These four therapies have many common methods and processes, including meta‐cognition, mindfulness, acceptance, decentering, self‐compassion, values‐focused behaviour, and perspective taking (Brown et al., 2011; Neff & Tirch, 2013).

The primary aim of this review was to use a meta‐analytic approach to determine the effectiveness of these four third wave interventions for children and adolescents for the following outcomes: (1) emotional symptoms/internalizing problems, (2) behavioural difficulties/externalizing problems, (3) interference from (emotional or physical) difficulties, (4) third wave processes (e.g. acceptance/mindfulness/self‐compassion), (5) well‐being/flourishing, (6) quality of life and (7) physical health/pain. The impact of study quality was also assessed. Secondary aims were to: (1) explore variation in effectiveness amongst types of third wave CBT, settings, populations, control conditions and formats of delivery (e.g. group versus individual therapy); (2) estimate effect sizes at follow‐up; and (3) compare third wave CBT to other psychological therapies. In keeping analyses comprehensive and broad, the review aimed to provide an initial, sweeping overview and synthesis of current data to inform future practice and research.

## METHOD

A protocol for this review was preregistered with PROSPERO (ID REMOVED FOR BLINDING).

### Literature search

PsycINFO, PubMed, Scopus and the Cochrane CENTRAL Trials Register were searched from inception to April 8th 2022 (initial searches were conducted in April 2019 before being updated in November 2019 then again in April 2022). The search strategy was: (“acceptance and commitment therapy” OR “compassion focus* therapy” OR “compassionate mind training” OR “mindfulness based cognitive therapy” OR “metacognitive therapy”) OR ([“third wave” OR “new wave”] AND therap*) AND (“child*” OR “adolescen*” OR “teen*” OR “parent*” OR “school” OR “youth*” OR “young people”). The first 200 results of both Google Scholar and a university library database were also searched.

Inclusion criteria comprised the following: (1) primary empirical studies that used a randomized controlled design (individual or cluster randomization); (2) investigating ACT, CFT, MBCT or MCT compared to a control group (which could be: no intervention, waitlist, or treatment as usual/an active intervention, as long as it was not one of the included third wave therapies); (3) with at least one outcome measure for children and adolescents under 18 years old; (4) that offered sufficient data in the paper (or by contacting authors) to calculate effect sizes required for meta‐analysis, of at least one outcome measure; and (5) were reported in an English language within a peer‐reviewed journal.

This review included studies conducted in any setting (e.g. schools, general hospitals or mental health clinics), using any measure related to the seven primary outcome domains (emotional symptoms/internalizing problems, behavioural difficulties/externalizing problems, interference from difficulties, third wave processes, wellbeing/flourishing, quality of life, and physical health/pain). The outcomes studied were intentionally broad given that the chosen forms of third wave CBT were promoted as transdiagnostic and relevant to thriving as well as pathology (Hayes & Hofmann, 2017).

Diagnosis/presentation or mode of delivery (e.g. face‐to‐face, online, telephone) did not serve as exclusion criteria. Interventions were included whether they were delivered to children and/or via parents/carers/significant others, as long as the child was the reason for accessing the intervention and there was a child‐focused outcome measure. In line with the protocol, non‐standardized interventions drawing on the four included therapies or combining other approaches were included, and accounted for within the quality assessment, to represent that many have attempt to implement third wave approaches to child populations in varying ways (Hayes & Ciarrochi, 2015).

### Eligible studies

**FIGURE 1:**
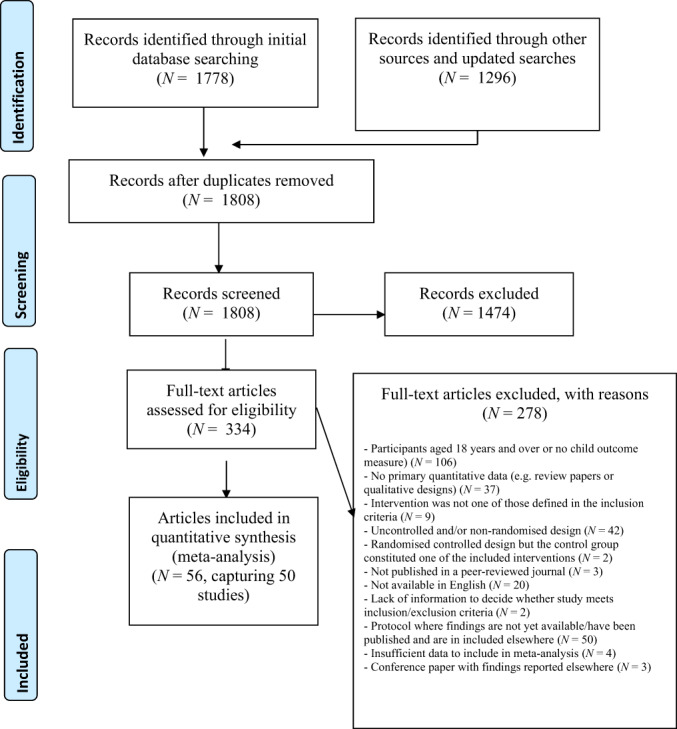
Diagram adapted from PRISMA, detailing flow of studies retrieved from searches through to inclusion.

The initial search produced 1778 results, plus there were an additional 400 records from alternative sources. Updated searches yielded a further 896 results. With duplicates removed, there were a total of 1808 records. The first author (AMP) screened titles and abstracts for eligibility. Full‐text articles of 334 potentially eligible studies were retrieved and further examined against inclusion and exclusion criteria by two authors (AMP, SWS). Any uncertainties regarding eligibility were resolved by discussion with a second or third reviewer (AGP, LP). Fifty‐six papers met inclusion criteria, describing 50 studies (Figure 1).

### Data extraction

Two authors (AMP, SWS) extracted demographic and methodological data. Data for meta‐analyses were also extracted following pre‐determined rules: (1) post‐intervention data were used in the primary analyses; (2) where there were multiple measures within a trial for a single outcome, the mean of effect sizes for all relevant measures was calculated and inputted into the meta‐analyses; (3) follow‐up data were extracted separately to post‐intervention data ‐ if multiple follow‐ups were completed, the furthest time point was chosen; (4) if there were multiple comparison groups, a non‐active control was chosen for the primary analyses in the first instance, followed by a non‐psychological then psychological intervention, given the primary research aim was to determine the effectiveness of third wave CBT not to compare it to other interventions (a separate, additional pool of data was extracted for secondary analyses comparing third wave CBT to other established psychological interventions); (5) data from intention‐to‐treat samples were included in analyses as preference, followed by data from subsets (e.g. assessment/treatment completers). All extraction of study characteristics and data for meta‐analyses were verified by a second reviewer.

### Quality assessment

Study quality was assessed using the Cochrane risk‐of‐bias tool for randomized trials (Sterne et al., 2019; Version 2), supplemented with items adapted from the NICE quality appraisal checklist for quantitative intervention studies (NICE, 2012). These additional items focused on quality of reporting, sampling (including generalizability and power), and the specificity (i.e. whether third wave CBT was distinct rather than combined with non‐relevant interventions, as described in inclusion criteria) and quality of the intervention (i.e. whether third wave CBT was manualized, comprehensive, developmentally adapted). The Cochrane tool for cluster‐randomized designs was used where appropriate (Eldridge et al., 2016). All papers were assigned quality ratings by two independent reviewers (AMP, SWS, AGP). The reviewers worked together if there were any ambiguities or discrepancies, to assign an agreed overall categorical rating of either low, moderate or high quality.

See Appendix S1 for the final quality ratings assigned to each study, and Appendix S2 for the quality assessment tool.

### Data analysis

Separate meta‐analyses were conducted to estimate effect sizes of third wave CBT at post‐intervention for each of the seven primary outcomes. The outcome categories were decided collaboratively by the research team prior to data extraction, based on clinical knowledge and literature (see Appendix S3). The impact of including low quality studies was assessed with sensitivity analyses. Secondary analyses were conducted to explore moderators, which included: (1) type of third wave CBT (ACT versus MBCT only, due to insufficient comparators for the other therapies); (2) setting (clinical [physical or mental health settings] versus non‐clinical [i.e. school or community, e.g. summer camps]); (3) type of control condition (active versus inactive); (4) participant age group (child versus adolescent); (5) delivery format (group versus individual therapy); and (6) parental involvement (child‐only versus joint/parent sessions). To correct for multiple comparisons within each meta‐analysis, the Holm‐Bonferroni method was used across moderators and subgroup analyses (Holm, 1979). Secondary analyses were also used to investigate effects at follow‐up and studies comparing third wave CBT to other psychological therapies.

Analyses were conducted using meta‐analysis via Shiny, which applies R programming language (MAVIS Version 1.1.3; Hamilton et al., 2017). Between‐group effect sizes were entered into random‐effects models to account for heterogeneity. MAVIS uses the metafor package (Viechtbauer, 2010) in R to calculate a standardized mean difference, which is converted to a Hedge's g (1981). Effect sizes of .2, .5 and .8 were interpreted as small, moderate and large, respectively (Fritz et al., 2012). The direction of effect which favoured third wave CBT was determined by the relative positivity or negativity of the scales which captured each outcome variable: for emotional symptoms/internalizing problems, behavioural difficulties/externalizing problems and interference from difficulties, a negative effect size favoured the intervention group whilst a positive effect size favoured the control comparison, whilst for third wave processes, wellbeing/flourishing, quality of life, and physical health/pain, a positive effect size favoured the intervention group whilst a negative effect favoured the control comparison.

*I* ^2^ was used to estimate the percentage of heterogeneity between studies that were not attributable to random sample error alone; values of 0%, 25%, 50% and 75% reflected nil, low, moderate and high levels of heterogeneity, respectively. Heterogeneity was also examined using the *Q‐*statistic; if significant (*p <* .05), it indicated that heterogeneity exceeded that expected by chance alone. Heterogeneity was subsequently explored using moderation and subgroup analyses. Moderation was tested using the *Qb* statistic, which is the level of variation explained by a covariate. Subgroup analyses were important to interpret any significant moderation effects, to address clinically important questions around what particular forms of third wave CBT might be effective, for whom, and in what settings. Subgroup analyses were still conducted when moderation was non‐significant, as high levels of heterogeneity within each subgroup could have led to a non‐significant moderation effect, even when subgroups vastly differed with regards to average effect size. To maintain reliability, moderation and subgroup analyses were not conducted if there were fewer than four studies per group.

Further sensitivity analyses excluded studies which used cluster randomization techniques. Funnel plots were created and publication bias was assessed in two steps. First, rank correlation tests for asymmetry were performed; a high and significant correlation (*p <* .05) indicated that the funnel plot was asymmetric and thus there was potential for bias. Second, visual inspection and trim‐and‐fill methods were used to estimate whether there were any missing studies that account for significant asymmetric distribution (Higgins & Green, 2011).

## RESULTS

### Sample size and characteristics

Fifty studies were included, comprising 4476 participants. Sample sizes ranged from 11–586, with a median of 50.5 (IQR 35.25–89.75). Studies were published between 2006 and 2022. Thirty‐three investigated ACT, 13 investigated MBCT, two investigated CFT approaches, one investigated MCT, and one investigated ACT combined with CFT. Twenty‐two studies utilized an inactive control group (no intervention/waitlist), 18 an active control group (namely treatment as usual or other interventions), and 10 made multiple comparisons (seven compared to both an inactive and active control group, and three compared to two different active conditions). Intervention duration ranged from a single, 30‐min session to 50 h of therapy over 20 weeks. Thirty‐four studies were group interventions, 14 comprised individual therapy, and two did not report delivery format. Thirty‐three delivered the intervention directly with the child/adolescent, eight with parent/carers only, and nine with both the parent/carer and young person.

Interventions were delivered in schools or communities (33), clinical physical health settings (11), and clinical mental health settings (6). Studies were conducted across various countries, including: Iran (17), Australia (8), Sweden (5), China (4), USA (4), Belgium (3), Finland (1), Denmark (1), Italy (1), Cyprus (1), Germany (1), Canada (1), India (1), Colombia (1) and the Philippines (1). Populations or conditions studied were related to: physical health (12), anxiety/stress (9), depression (4), family/care situations (4), behavioural difficulties (3), learning/neurodevelopmental difficulties (2), social/school difficulties (2), trichotillomania (2), eating/body image disorders (2), low wellbeing (1), and mixed internalizing and/or externalizing difficulties (2). The remaining studies (7) were conducted with general samples (e.g. exploring interventions as preventative or promotive wellbeing strategies). The age of participants ranged from 0–18 (*M* = 12.92*, SD* = 3.27); although, these statistics do not include nine studies in which average age was unreported. Twenty‐three studies collected follow‐up data in addition to post‐intervention effects, with three studies excluded from analysis at this point due to no longer meeting inclusion criteria; follow‐up length ranged from 1–24 months (*Mdn* = 3.5, IQR 2–6).

Appendix S1 further details the characteristics of included studies.

### Study quality and attrition

Ten studies were rated as high quality, 22 moderate quality, and 18 low quality. Twelve studies were rated highly with regards to quality of the intervention (Appendix S1). Eleven studies did not report sample attrition at post‐treatment; of those that did, dropout rates ranged from 0–61.54%, with a median of 9.64% (IQR 4.33–19.07). Median attrition at furthest follow‐up was 20.29% (IQR 14.95–38.69); though this excludes four studies that did not report dropout. Sixteen studies did not specify whether data represented all participants randomized at outset or only a subset (e.g. assessment/treatment completers); for these papers, it was assumed that data represented the completer sample for each time point. Eight studies specified using a completer sample. For 20 studies, it was reported that intention‐to‐treat methods were applied (at least partially or in some form to include all participants randomized). In six studies, there was no attrition and thus a complete sample.

### Primary analyses

Main effects for the primary outcomes at post‐intervention are presented in Table 1. Twenty‐nine studies at post‐intervention compared to inactive controls while the remainder used active conditions (see Table 1 for a breakdown per outcome). These active comparisons included: other psychological interventions (5), broad “treatments as usual”, such as multi‐disciplinary care (5), school pastoral/counsellor/nurse support (4), educational/activity‐based groups (4), medication/medical care (2) and the usual school curriculum (1). Overall, significant and small sized effects were found favouring third wave CBT for interference from difficulties and third wave processes. Significant medium‐sized superiority effects were found for emotional symptoms/internalizing problems, behavioural difficulties/externalizing problems, wellbeing/flourishing, and physical health/pain. A non‐significant effect was only observed for quality of life. For all variables, there was significant heterogeneity (*I* ^2^ ranged from 79%–97%).

**TABLE 1:** Main effects for the primary outcome variables

	*k*	*g* ^a^	95% CI	*p*‐value	Heterogeneity *I* ^2^ (*Q* with *p*‐value)
Emotional symptoms and internalizing problems (*A* = 19, *I* = 24)					
Overall effect (*N* = 3265)	**43**	**−.68**	**−.98 to −.37**	**<.001**	94% (435.30, <.001)
Excluding low quality studies (*N* = 2110)	**28**	**−.55**	**−.82 to −.27**	**<.001**	88% (132.78, <.001)
Behavioural difficulties and externalizing problems (*A* = 9, *I* = 14)					
Overall effect (*N* = 1659)	**23**	**−.62**	**−1.01 to −.22**	**.002**	93% (168.12, <.001)
Excluding low quality studies (*N* = 1351)	15	−.38	−.86 to .10	.119	94% (107.48, <.001)
Interference from difficulties (*A* = 13, *I* = 8)					
Overall effect (*N* = 1786)	**21**	**−.46**	**−.87 to −.05**	**.028**	93% (267.99, <.001)
Excluding low quality studies (*N* = 1605)	**17**	**−.48**	**−.90 to −.05**	**.027**	93% (213.17, <.001)
Third wave processes (*A* = 11, I = 11)					
Overall effect (*N* = 1900)	**22**	**.39**	**.17 to .62**	**<.001**	79% (79.20, <.001)
Excluding low quality studies (*N* = 1692)	**18**	**.27**	**.11 to .43**	**<.001**	51% (33.90, .009)
Wellbeing and flourishing (*A* = 8, *I* = 13)					
Overall effect (*N* = 1303)	**21**	**.76**	**.35 to 1.17**	**<.001**	92% (146.42, <.001)
Excluding low quality studies (*N* = 1063)	**16**	**.50**	**.18 to .81**	**.002**	82% (66.56, <.001)
Quality of life (*A* = 5, *I* = 7)					
Overall effect (*N* = 1271)	12	.62	−.08 to 1.31	.082	97% (84.89, <.001)
Excluding low quality studies (*N* = 1212)	**10**	**.32**	**.04 to .60**	**.024**	77% (34.83, <.001)
Physical health and pain (*A* = 5, *I* = 4)					
Overall effect (*N* = 1171)	**9**	**.72**	**.01 to 1.44**	**.047**	96% (163.38, <.001)
Excluding low quality studies (*N* = 1139)		.52	−.14 to 1.17	.122	96% (141.38, <.001)

Abbreviations: *A*, number of active controls in the main analysis for overall effect; CI, confidence interval; *I*, number of inactive controls in the main analysis for overall effect; *I* ^2^, percentage heterogeneity; *k*, number of studies; *g*, Hedges' *g*; *N*, participants included in analysis (based on intention‐to‐treat sample where available); *Qb*, *Qb*‐statistic or level of variation explained by a covariate.

^a^ For emotional symptoms/internalizing problems, behavioural difficulties/externalizing problems, and interference from difficulties, a negative effect size favours the intervention group whilst a positive effect size favours the control comparison. For the remaining outcomes (third wave processes, well‐being/flourishing, quality of life and physical health/pain), a positive effect size favours the intervention group whilst a negative effect favours the control comparison.

Significant effect sizes (*p* < .05) are denoted in bold.

### Impact of study quality

Sensitivity analyses excluding low quality studies were performed and are presented alongside main effects in Table 1. Results indicated that study quality had a substantial impact for some outcome variables. Behavioural difficulties/externalizing problems and physical health/pain ceased to be significant. Quality of life now yielded a small significant effect. Emotional symptoms/internalizing problems and wellbeing/flourishing remained significant with a moderate effect size, whilst interference from difficulties and third wave processes remained significant with small effect sizes.

**FIGURE 2:**
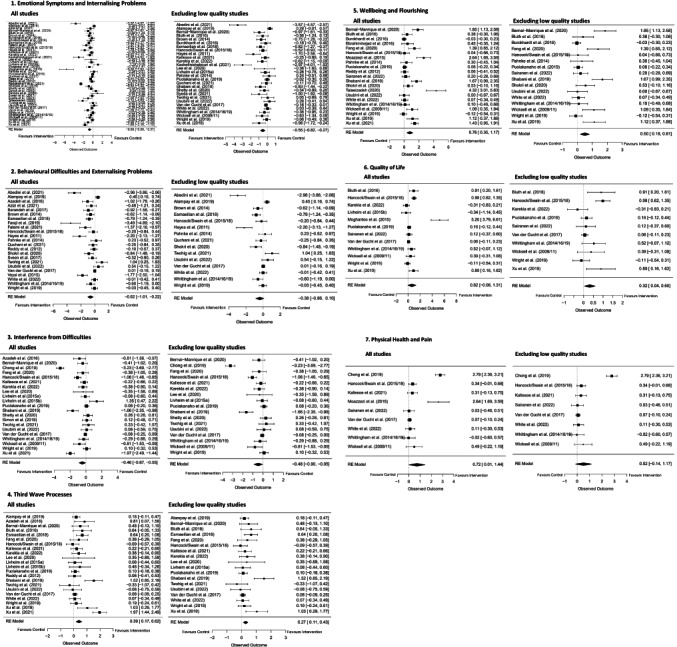
Forest plots detailing effect sizes with 95% confidence intervals for the seven primary outcome variables, inclusive and exclusive of low quality studies.

Figure 2 depicts forest plots for the primary analyses.

### Secondary analyses

#### Moderation and subgroup analyses

Moderation with subgroup analyses are presented to aid interpretation of heterogeneity (Table 2). However, these should be interpreted with caution as studies rated as low quality were included to maintain number of available comparators. There remained too few comparators for some moderator analyses (<4 per subgroup) as indicated on Table 2. All studies included in analyses for physical health/pain used ACT interventions. No significant moderators were found for any of the seven primary outcomes, with the exception of control condition for emotional symptoms/internalizing problems, with third wave CBT yielding a large and significant effect compared to inactive controls but a small and non‐significant effect compared to active controls. As detailed in Table 3, analyses yielded some other interesting subgroup results (e.g. relating to participant age, type of third wave CBT, involvement of parents/carers in sessions, whether sessions were delivered in groups or to individuals, and whether studies were conducted in clinical or community settings); however, there were no clear patterns across variables and these analyses should be interpreted in context of moderation analyses remaining non‐significant as well as other limitations such as the small number of studies within selected subgroups.

**TABLE 2:** Moderation and subgroup analyses for the primary outcome variables

	*k*	*g* ^a^	95% CI	*p*‐value	Heterogeneity *I* ^2^ (*Q* with *p*‐value)
Emotional symptoms and internalizing problems					
Intervention type (*Qb* = .98, *p* = .322)					
ACT	**28**	**−.63**	**−.95 to −.30**	**<.001**	92% (330.60, <.001)
MBCT	**11**	**−.93**	**−1.45 to −.42**	**<.001**	90% (97.25, <.001)
Setting (*Qb* = .50, *p* = .480)					
Clinical	**15**	**−.79**	**−1.22 to −.35**	**<.001**	87% (108.23, <.001)
Non‐clinical	**28**	**−.59**	**−.90 to −.29**	**<.001**	92% (326.13, <.001)
Control condition (*Qb* = 8.93, *p* = .003)					
Active	19	−.27	−.61 to .07	.116	82% (99.26, <.001)
Inactive	**24**	**−.96**	**−1.27 to −.66**	**<.001**	90% (237.76, <.001)
Delivery (*Qb* = 1.37, *p* = .242)					
Individual	13	−.41	−.88 to .06	.085	78% (53.42, <.001)
Group	**28**	**−.75**	**−1.06 to −.44**	**<.001**	93% (369.90, <.001)
Parental involvement (*Qb* = 2.75, *p* = .098)					
Child‐only	**29**	**−.80**	**−1.11 to −.50**	**<.001**	93% (417.01, <.001)
Parents involved	14	−.35	−.79 to .10	.125	20% (16.19, .239)
Participant age (*Qb* = .18, *p* = .674)					
Child	6	−.49	−1.13 to .15	.131	0% (4.14, .530)
Adolescent	**22**	**−.65**	**−.98 to −.32**	**<.001**	92% (270.80, <.001)
Behavioural difficulties and externalizing problems					
Intervention type (*Qb* = 3.85, *p* = .050*)					
ACT	14	−.38	−.81 to .04	.079	79% (61.33, <.001)
MBCT	**7**	**−1.13**	**−1.74 to −.52**	**<.001**	94% (103.71, <.001)
Setting (*Qb* = .01, *p* = .919)					
Clinical	9	−.56	−1.06 to −.07	.027*	88% (67.28, <.001)
Non‐clinical	**14**	**−.60**	**−.99 to −.20**	**.003**	86% (95.18, <.001)
Control condition (*Qb* = .60, *p* = .438)					
Active	9	−.44	−.89 to .02	.060	90% (80.67, <.001)
Inactive	**14**	**−.67**	**−1.06 to −.29**	**<.001**	79% (61.48, <.001)
Delivery (*Qb* = 1.13, *p* = .289)					
Individual	4	−.18	−.94 to .58	.641	90% (31.57, <.001)
Group	**17**	**−.64**	**−.99 to −.28**	**<.001**	87% (127.37, <.001)
Parental involvement (*Qb* = 2.66, *p* = .103)					
Child‐only	**14**	**−.80**	−**1.21 to −.39**	**<.001**	91% (149.46, <.001)
Parents involved	9	−.27	−.76 to .23	.293	56% (18.02, .021)
Participant age (*Qb* = .47, *p* = .494)					
Child	6	−.85	−1.43 to −.26	.004*	73% (18.32, .003)
Adolescent	10	−.59	−1.03 to −.15	.008*	86% (65.68, <.001)
Interference from difficulties					
Intervention type					
ACT	–	–	–	–	–
MBCT	–	–	–	–	–
Setting (*Qb* = 3.37, *p* = .066)					
Clinical	**8**	**−.88**	**−1.45 to −.31**	**.002**	95% (134.69, <.001)
Non‐clinical	13	−.20	−.65 to .25	.376	83% (70.14, <.001)
Control condition (*Qb* = .49, *p* = .484)					
Active	13	−.36	−.84 to .13	.151	94% (212.06, <.001)
Inactive	8	−.64	−1.27 to −.01	.048*	81% (36.76, <.001)
Delivery (*Qb* = 1.34, *p* = .247)					
Individual	7	−.12	−.82 to .58	.733	28% (8.29, .218)
Group	14	−.62	−1.10 to −.14	.011*	95% (245.40, <.001)
Parental involvement (*Qb* = 1.94, *p* = .164)					
Child‐only	12	−.24	−.72 to .25	.336	84% (67.04, <.001)
Parents involved	9	−.77	−1.34 to −.20	.008*	95% (158.56, <.001)
Participant age					
Child	–	–	–	–	–
Adolescent	–	–	–	–	–
Third wave processes					
Intervention type (*Qb* = .07, *p* = .785)					
ACT	**16**	**.38**	**.13 to .63**	**.003**	78% (69.75, <.001)
MBCT	4	.46	−.02 to .93	.058	56% (6.80, .079)
Setting (*Qb* = .55, *p* = .457)					
Clinical	5	.24	−.19 to .68	.275	79% (19.09, <.001)
Non‐clinical	**17**	**.43**	**.20 to .66**	**<.001**	73% (59.97, <.001)
Control condition (*Qb* = 2.39, *p* = .122)					
Active	11	.25	−.00 to .50	.053	47% (18.87, .042)
Inactive	**11**	**.55**	**.27 to .83**	**<.001**	79% (47.40, <.001)
Delivery (*Qb* = .79, *p* = .373)					
Individual	6	.22	−.20 to .64	.297	40% (8.33, .139)
Group	**16**	**.44**	**.21 to .68**	**<.001**	79% (70.09, <.001)
Parental involvement (*Qb* = .15, *p* = .695)					
Child‐only	**17**	**.41**	**.18 to .63**	**<.001**	74% (60.88, <.001)
Parents involved	5	.31	−.15 to 76	.188	78% (18.32, .001)
Participant age					
Child	–	–	–	–	–
Adolescent	–	–	–	–	–
Wellbeing and flourishing					
ACT	**15**	**.88**	**.47 to 1.30**	**<.001**	90% (134.78, <.001)
MBCT	4	.47	−.33 to 1.28	.245	66% (8.95, .030)
Setting (*Qb* = .02, *p* = .899)					
Clinical	7	.76	.18 to 1.34	.010*	85% (39.97, <.001)
Non‐clinical	**14**	**.72**	**.31 to 1.12**	**<.001**	87% (103.25, <001)
Control condition (*Qb* = 1.77, *p* = .183)					
Active	8	.46	−.03 to .96	.065	82% (39.99, <.001)
Inactive	**13**	**.90**	**.49 to 1.31**	**<.001**	86% (86.91, <.001)
Delivery (*Qb* = 2.27, *p* = .132)					
Individual	**7**	**1.12**	**.52 to 1.73**	**<.001**	91% (70.42, <.001)
Group	14	.56	.15 to .97	.008*	83% (75.47, <.001)
Parental involvement (*Qb* = 1.13, *p* = .288)					
Child‐only	**14**	**.86**	**.45 to 1.28**	**<.001**	89% (121.26, <.001)
Parents involved	7	.48	−.09 to 1.05	.100	76% (25.02, <.001)
Participant age					
Child	–	–	–	–	–
Adolescent	–	–	–	–	–
Quality of life					
Intervention type					
ACT	–	–	–	–	–
MBCT	–	–	–	–	–
Setting (*Qb* = 5.42, *p* = .020*)					
Clinical	**5**	**.98**	**.44 to 1.52**	**<.001**	92% (48.20, <.001)
Non‐clinical	7	.15	−.28 to .59	.484	58% (14.28, .027)
Control condition (*Qb* = 5.06, *p* = .025*)					
Active	5	.04	−.48 to .56	.885	0% (2.89, .576)
Inactive	7	**.84**	**.37 to 1.31**	**<.001**	90% (62.07, <.001)
Delivery (*Qb* = 1.48, *p* = .224)					
Individual	5	.23	−.39 to .84	.470	46% (7.35, .118)
Group	7	.73	.20 to 1.26	.007*	92% (76.77, <.001)
Parental involvement (*Qb* = .30, *p* = .583)					
Child‐only	7	.60	.08 to 1.11	.023*	91% (63.27, <.001)
Parents involved	5	.38	−.20 to .96	.198	76% (16.85, .002)
Participant age					
Child	–	–	–	–	–
Adolescent	–	–	–	–	–
Physical health and pain					
Intervention type					
ACT	–	–	–	–	–
MBCT	–	–	–	–	–
Setting					
Clinical	–	–	–	–	–
Non‐clinical	–	–	–	–	–
Control condition (*Qb* = .01, *p* = .918)					
Active	5	.75	−.16 to 1.67	.105	97% (137.58, <.001)
Inactive	4	.68	−.36 to 1.72	.199	88% (25.67, <.001)
Delivery					
Individual	–	–	–	–	–
Group	–	–	–	–	–
Parental involvement (*Qb* = .00, *p* = .957)					
Child‐only	4	.70	−.27 to 1.67	.159	89% (27.80, <.001)
Parents involved	5	.73	−.13 to 1.60	.097	96% (109.03, <.001)
Participant age					
Child	–	–	–	–	–
Adolescent	–	–	–	–	–

Notes

Where “−” is observed, moderation and subgroup analyses were not possible due to an insufficient number of studies (<4) per subgroup. Significant moderators and subgroups are denoted in bold.

Abbreviations: ACT, Acceptance and Commitment Therapy; CI, confidence interval; *g*, Hedges' *g*; *I* ^2^, percentage heterogeneity; *k*, number of studies in subgroup; MBCT, Mindfulness‐Based Cognitive Therapy; *Qb*, *Qb*‐statistic or level of variation explained by a covariate.

^a^ For emotional symptoms/internalizing problems, behavioural difficulties/externalizing problems, and interference from difficulties, a negative effect size favours the intervention group whilst a positive effect size favours the control comparison. For the remaining outcomes (third wave processes, wellbeing/flourishing, quality of life, and physical health/pain), a positive effect size favours the intervention group whilst a negative effect favours the control comparison.

*Whilst *p* **< .**05, this was non‐significant following correction using the Holm‐Bonferroni method.

**TABLE 3:** Meta‐analyses of follow‐up data

	*k*	*g* ^a^	95% CI	*p*‐value	Heterogeneity *I* ^2^ (*Q* with *p*‐value)
Emotional symptoms and internalizing problems (*A* = 8, *I* = 9)					
Overall effect (*N* = 1680)	**17**	**−.76**	**−1.32 to −.20**	**.008**	96% (133.31, <.001)
Excluding low quality studies (*N* = 1242)	**13**	**−.91**	**−1.64 to −.18**	**.014**	97% (121.64, <.001)
Behavioural difficulties and externalizing problems (*A* = 6, *I* = 5)					
Overall effect (*N* = 902)	**11**	**−.83**	**−1.51 to −.15**	**.016**	95% (106.52, <.001)
Excluding low quality studies (*N* = 789)	8	−.90	−1.88 to .07	.068	97% (88.86, <.001)
Interference from difficulties^b^ (*A* = 6, *I* = 1)					
Overall effect (*N* = 846)	7	−1.33	−2.85 to .19	.086	99% (281.54, <.001)
Third wave processes^b^ (*A* = 4, *I* = 2)					
Overall effect (*N* = 821)	6	.58	−.28 to 1.44	.188	97% (46.22, <.001)
Wellbeing and flourishing^b^ (*A* = 5, *I* = 2)					
Overall effect (*N* = 433)	7	.41	−.20 to 1.02	.188	87% (30.35, <.001)
Quality of life^‡^ (*A* = 4, *I* = 1)					
Overall effect (*N* = 642)	5	.14	−.01 to .30	.069	0% (1.41, .843)
Physical health and pain^b^ (*A* = 5, *I* = 2)					
Overall effect (*N* = 895)	7	.64	−.50 to 1.78	.269	98% (212.90, <.001)

Abbreviations: *A*, number of active controls in the main analysis for overall effect; CI, confidence interval; *g*, Hedges' *g*; *I*, number of inactive controls in the main analysis for overall effect; *I* ^2^, percentage heterogeneity; *k*, number of studies; *N*, participants included in analysis (based on intention‐to‐treat sample where available); *Qb*, *Qb*‐statistic or level of variation explained by a covariate.

^a^ For emotional symptoms/internalizing problems, behavioural difficulties/externalizing problems, and interference from difficulties, a negative effect size favours the intervention group whilst a positive effect size favours the control comparison. For the remaining outcomes (third wave processes, wellbeing/flourishing, quality of life, and physical health/pain), a positive effect size favours the intervention group whilst a negative effect favours the control comparison.

^b^ All studies at follow‐up were rated moderate‐high quality.

Significant effect sizes (*p* < .05) are denoted in bold.

#### Effects at follow‐up

Main effects at follow‐up are presented in Table 3. There were a relatively limited number of studies with follow‐up data, particularly for interference from difficulties, third wave processes, wellbeing/flourishing, quality of life, and physical health/pain. A significant overall effect favouring third wave CBT was observed for emotional symptoms/internalizing problems, which was moderate in size with all studies included and large with low quality studies excluded. For behavioural difficulties/externalizing problems, a large and significant effect was found with all studies included; however, this became non‐significant with low quality studies excluded. No significant effects were found for the other variables. There was significant heterogeneity for six of seven outcome variables.

#### Comparison to other psychological therapies

Eleven studies had a control group comprising a specific other psychological intervention, which were: conventional CBT (3), conventional CBT plus SSRI medication (1), conventional CBT (plus family therapy for one participant) (1); cognitive restructuring from conventional CBT (1), narrative exposure and response prevention (1), cognitive emotion regulation protocol (1), emotion regulation training (1), reality therapy based on choice theory (1) and the Stepping Stones Triple P Programme (1). For emotional symptoms/internalizing problems, there was a non‐significant, negligible difference to other psychological therapies (*k* = 10, *g* = −.17, 95% CI −.62 to .29, *p* = .472). For behavioural difficulties/externalizing problems, there was a small but non‐significant difference favouring third wave CBT (*k* = 6, *g =* −.48, 95% CI −1.14 to .19, *p* = .161). For interference from difficulties, there was a negligible, non‐significant difference between groups (*k =* 5, *g =* −.11, 95% CI −.34 to .11, *p* = .309). For wellbeing/flourishing, there was a small but non‐significant difference favouring third wave CBT (*k =* 6, *g =* .37, 95% CI −.12 to .86, *p* = .139). There were too few comparisons to explore the other outcome variables.

### Further sensitivity analyses and publication bias

With studies using cluster randomization techniques excluded, results were comparable to main effects (Appendix S4). However, significant effects for wellbeing/flourishing and physical health/pain with all studies included were recategorized as large rather than moderate.

Rank correlation tests for funnel plot asymmetry were non‐significant for four of the seven primary outcomes; however, those which were significant included emotional difficulties/internalizing problems, behavioural difficulties/externalizing problems and well‐being/flourishing (Appendix S5). Nonetheless, visual analysis of funnel plots for these significant outcomes was not suggestive of bias and no missing studies were estimated. Whilst it was estimated that there were five missing null studies for interference from difficulties, asymmetry was non‐significant. See Appendix S4 for rank correlation tests and funnel plots.

## DISCUSSION

### Main findings

Fifty RCTs were included in this meta‐analysis. Main analyses yielded significant small‐moderate effects at post‐treatment in favour of third wave CBT compared to control conditions (both active/inactive) for measures of emotional symptoms/internalizing problems, behavioural difficulties/externalizing problems, interference from difficulties, third wave processes and wellbeing/flourishing, alongside non‐significant findings for quality of life. Nonetheless, a number of the identified studies were rated as low quality. It was decided a priori to consider the impact of study quality and other study characteristics, given the application of third wave CBT to child and adolescent populations is relatively novel and has been done with great variation. Sensitivity analyses excluding low quality studies changed results for some outcomes; behavioural difficulties/externalizing problems ceased to be significant whilst quality of life then yielded a small significant effect in favour of third wave CBT.

There were high levels of significant heterogeneity for all outcomes. Despite moderation analyses, this heterogeneity remained largely unexplained. Type of third wave CBT, setting, group versus individual delivery, parental involvement in therapy and participant age were not found to be significant moderators for any outcome. Type of control comparison (i.e. active versus inactive) was only a significant moderator for emotional symptoms/internalizing problems. Additional subgroup analyses yielded some interesting results however should be interpreted in context of moderation analyses remaining non‐significant. The reliability and validity of any significant moderators or apparent subgroup differences also needs to be carefully considered, given that low quality studies were included in these analyses; thus, effects may be explained by quality rather than the moderator or subgroup variable itself.

At follow‐up, moderate‐large significant superiority effects for third wave CBT were observed for emotional symptoms/internalizing problems and behavioural difficulties/externalizing problems. When low quality studies were excluded, the effect for behavioural difficulties/externalizing problems became non‐significant whilst the effect for emotional symptoms/internalizing problems remained significant and became large in size. For the remaining outcome variables, no significant effects were found at follow‐up, with or without low quality studies included. Nonetheless, given that only a minority of trials at post‐treatment included follow‐up assessments, it was difficult to evaluate maintenance effects. Power could have been limited, and there was a differing composition of studies for post‐intervention compared to follow‐up. For example, at follow‐up for interference from difficulties, third wave processes, quality of life, wellbeing/flourishing and physical health/pain, a greater proportion of studies utilized active control groups, relative to those studies that comprised post‐treatment comparisons. Another difference was that for these variables, many studies at follow‐up were conducted in clinical settings, whereas most studies at post‐treatment were conducted in non‐clinical settings. Analyses specifically comparing third wave to other psychological therapies at post‐treatment showed no significant differences.

### Clinical and research implications

It is essential to ensure the quality of interventions offered within child and adolescent services, as well as investigate universal approaches that can be used more widely as preventative and promotive public health strategies for youth (DoH & DfE, 2017; PHE, 2019). Until now, no meta‐analysis existed to determine the effectiveness of these four types of third wave CBT as a transdiagnostic approach for young people, despite their arguable applicability across presentations, along the spectrum from ill‐health to flourishing, and subsequent popularity within clinical and non‐clinical settings. Overall, the present results suggest that third wave CBT is a promising intervention. Significant post‐treatment effects were found across a range of outcomes from symptomatology (such as emotional symptoms and internalizing problems) to thriving (such as outcomes capturing wellbeing, flourishing and quality of life). Sample size remained high for sensitivity analyses considering study quality, increasing confidence in the findings.

A significant effect was found for interference from difficulties. This pattern fits with the premise of third wave CBT, which often places more emphasis on facilitating change by altering a person's *relationship* with thoughts, emotions or difficulties (Hayes, 2004). Third wave processes also yielded significant effects, supporting change amongst underlying mechanisms of action targeted by these types of third wave CBT for children and adolescents. Quality‐controlled analyses suggested that third wave CBT may not be effective for behavioural difficulties/externalizing problems and physical health/pain. It is however important to note that there was considerable heterogeneity for both of these outcomes, with small‐moderate overall effect sizes favouring third wave CBT; this heterogeneity suggests that conclusions may not be generalizable and that further investigation is warranted.

Whilst comparisons were limited, findings suggested that third wave CBT did not perform significantly differently from other psychological therapies specifically, which included conventional CBT. At present, second wave CBT is viewed as the “gold standard” treatment in clinical guidelines (David et al., 2018). This review indicates that third wave interventions could be similarly effective, though this requires further research as only four of the seven primary outcomes could be compared to other psychological therapies and study numbers were limited. Cost‐effectiveness reporting and analyses are also essential in future research; the majority of included studies conducted relatively short, group interventions, suggesting the possibility that third wave CBT may be an inexpensive, clinically effective alternative to current treatments.

Maintenance effects were also difficult to evaluate as a relatively small number of studies included follow‐up data compared to post‐treatment. It is important to note that there was limited evidence that third wave CBT is effective for inducing long‐term change, except amongst the category of emotional symptoms/internalizing problems. Also promising was that despite being non‐significant, effect sizes were moderate‐large in favour of third wave CBT for behavioural difficulties/externalizing problems, interference from difficulties, third wave processes and quality of life. More high‐quality RCTs are needed to increase the scope and power of any future meta‐analyses assessing follow‐up effects. This is important to know whether such interventions are worth investing in to improve long‐term outcomes. Similarly, for earlier generations of CBT for children and adolescents, follow‐up effects are often not explored or are non‐significant (Battagliese et al., 2015; James et al., 2015; Uhre et al., 2020) and thus this could be a widespread issue for clinical consideration.

Despite the promising overall findings of this review, it is worth noting that widespread heterogeneity raised queries about generalizability, with moderation analyses offering limited explanation. Further high‐quality trials would also allow further investigation into potential moderator and subgroup effects which were preliminary explored in this review, with many planned analyses not being possible to conduct due to limited study numbers. When research is available, it is important to also explore moderator and subgroup analyses in conjunction as they may account for one another; for example, any differences between clinical and non‐clinical settings could be explained by mode of delivery in terms of group versus individual therapy. Similarly, there may be other important moderators to consider which were not included in this review, such as whether third wave CBT is used as a treatment, preventative, or promotive health intervention. There remains unexplained heterogeneity amongst findings from this analysis.

### Strengths and limitations

This was a comprehensive review including a range of outcomes and a high number of participants across both clinical and non‐clinical settings. It enabled a thorough investigation into the effectiveness of specific types of third wave CBT for children and adolescents, from the treatment of symptomatology to promotion of thriving, as well as a rigorous evaluation of study quality. It is recognized that the wide variety of studies included means it is not possible to determine effectiveness for specific groups or situations; however, this review aimed to take a broad and exploratory stance into a relatively novel, transdiagnostic approach for supporting child and adolescent mental health and wellbeing.

It is important that future research gives due attention to quality considerations. For example, for 24 of the included studies, it was specified or assumed that data represented completer samples only. Analysing only a subset of those randomized can lead to overestimation of effect sizes, however. For example, because drop out may occur when participants find an intervention unhelpful or unacceptable (Gupta, 2011). This should be held in mind when interpreting results from this meta‐analysis, despite capturing use of intention‐to‐treat methods within quality ratings. Similarly, only 10 studies were rated as high quality overall, and only seven of these scored highly with regards to the specificity and quality of the intervention, making it further difficult to evaluate third wave CBT. Several papers used interventions that were unstandardized or otherwise limited (e.g. exploring only defusion from ACT). If high‐quality interventions were delivered, effects at post‐treatment and follow‐up may have differed.

It is important to note that an a priori power analysis was not undertaken. No meta‐analysis had been conducted to examine the four included types of third wave CBT for children and adolescents when this review began, and results from individual trials had been varied. Thus, expected effect sizes were unknown and informed power calculations would have been difficult. Regardless of how well powered a meta‐analysis is, such an approach is still the optimal strategy for synthesizing available literature, compared to other methods available (Valentine et al., 2010). Nonetheless, where between‐study variance is high, higher power is needed to detect effects. It should be recognized that power may have been limited for some analyses, particularly where study numbers were low (e.g. at follow‐up/within subgroup analyses).

A further limitation of this review was that, given there is no definitive list or agreement about which interventions constitute ‘third wave CBT’, decisions around which therapies were included and excluded will be open to question. It should be recognized that conclusions from this review are limited to only those therapies included. For example, dialectical behaviour therapy (Linehan, 1993) and schema therapy (Young, 1990) were amongst those excluded for their focus on axis I disorders, and mindfulness‐based stress reduction (Kabat‐Zinn, 1982) for its limited use of cognitive behavioural techniques beyond mindful meditation. In future research, it is important to explore other third wave interventions and compare them to those included in this review as well as earlier generations of therapy.

## CONCLUSIONS

To our knowledge, this is the first meta‐analysis to consider the effectiveness of several types of third wave CBT as a transdiagnostic group of therapies for children and adolescents: specifically, ACT, CFT, MBCT and MCT for the scope of this review. Fifty RCTs were identified, though many were of poor quality, both with regards to research design and the intervention delivered. Results were promising; when low quality studies were excluded, significant effects were found for a variety of outcomes, including emotional symptoms/internalizing problems, interference from difficulties, third wave processes, well‐being/flourishing and quality of life. The results were non‐significant for behavioural difficulties/externalizing problems and physical health/pain. However, widespread heterogeneity remained for all variables, raising queries about the generalizability of findings. There was only significant evidence that third wave CBT is effective for inducing long‐term change for emotional symptoms/internalizing problems. Whilst further high‐quality research is warranted, including to explore heterogeneity and maintenance effects, this review indicates that third wave CBT could be a beneficial transdiagnostic approach to treatment, as well as a public health tool to promote thriving, amongst young people.

## AUTHOR CONTRIBUTIONS

**Samuel W. Spaul:** Data curation; formal analysis; validation; writing – review and editing. **Gemma Bowers:** Conceptualization; methodology; supervision; validation; writing – review and editing. **Abigail Perkins:** Formal analysis; methodology; validation; writing – review and editing. **Laura Pass:** Formal analysis; methodology; supervision; validation; visualization; writing – review and editing.

## FUNDING INFORMATION

The authors did not receive any specific funding or grants from any organization for the submitted work.

## CONFLICT OF INTEREST

The authors have no relevant financial or non‐financial interests to disclose.

## Supporting information


Appendix S1:

## Data Availability

The data that support the findings of this study are available from the corresponding authors upon reasonable request.
