# Supplementary material for: The effectiveness of third wave cognitive behavioural therapies for children and adolescents: A systematic review and meta‐analysis
Source: Br J Clin Psychol. 2022 Nov 28;62(1):209–27. doi: 10.1111/bjc.12404 (PMC10100516; doi:10.1111/bjc.12404)
Supplement: Supplementary file 1 — Appendix S1: [file BJC-62-209-s001.docx]

Supplementary Material A: Table of Included Studies

| Study author, year*** | Intervention, duration (mins per session), level of child-parent involvement, and delivery format | Conditions | Participant group† (M age) and target condition | N (% dropout at post-treatment); ITT or subset sample | Setting and country | Longest follow-up in months (with % drop out at furthest time) | Quality rating |  |
| --- | --- | --- | --- | --- | --- | --- | --- | --- |
| Abedini, Habibi, Abedini, Achenbach & Semple (2020)** | MBCT; 20 sessions over 4 weeks (45); Child-only; Group Sessions | 1. MBCT  2. TAU | Children (12.12); Cancer with emotional/attentional difficulties | 40 (2.5); ITT | Hospital, Iran | 2 (17.5) | High | |
|  |  |  |  |  |  |  |  |  |
| Alampay et al. (2019) | MBCT; 8 weekly sessions (75-90); Child-only; Group sessions | 1. MBCT  2. Active control (handicrafts) | Children and adolescents (11.88); Behavioural, emotional or peer difficulties | 186 (11.83); ITT | School, Philippines | 2 (21.51) | Moderate |  |
| Azadeh, Kazemi-Zahrani & Besharat (2016) | ACT; 10 weekly sessions (90); Child-only; Group sessions | 1. ACT  2. No intervention/waitlist | Adolescents (15.43); Social anxiety disorder | 30 (NR); NR | School, Iran | NA | Low |  |
| Azizi, Sepehri & Demehri (2021) | ACT combined with CFT; 8 weekly sessions (90); Parent-only; NR | 1. ACT  2. No treatment | Children (NR); hearing impairment | 30 (NR); NR | School, Iran | NA | Low | |
|  |  |  |  |  |  |  |  |  |
| Barandeh, Shafiabadi & Farzad (2017) | ACT; 8 weekly sessions (NR); Child-only; Group sessions | 1. ACT  2. Choice theory intervention  3. No intervention/waitlist | Adolescents (NR); Procrastination difficulties | 60 (NR); NR | School, Iran | 1 (NR) | Low |  |
| Bernal-Manrique, Garcia-Martin & Ruiz (2020) | ACT: 3 weekly sessions (75); Child-only; Group Sessions | 1. ACT  2. Waitlist | Adolescents (14.52); social and school difficulties | 42 (0); NA | School, Colombia | NA | Moderate | |
|  |  |  |  |  |  |  |  |  |
| Bluth, Gaylord, Campo, Mullarkey & Hobbs (2016) | CFT; 6 weekly sessions (90); Child-only; Group sessions | 1. Mindful Self-Compassion Program  2. No intervention/ waitlist | Adolescents (NR); General population | 34 (14.71); NR | School/community, USA | NA | Moderate |  |
| Brown, Whittingham, Boyd, McKinlay, & Sofronoff (2014) | ACT; 2 sessions (120) plus 9 SSTP sessions; Parent-only; Group sessions | 1. ACT + SSTP  2. TAU | Child (7.00); Acquired brain injury | 59 (11.86); ITT | Clinical PH, Australia | 6 (47.46) -but no longer met inclusion criteria so exclude from analyses | Moderate |  |
| Burckhardt, Manicavasgar, Batterham & Hadzi-Pavlovic (2016) * | ACT; 16 sessions over 3 months (30); Child-only; Group sessions | 1. ACT (+ positive psychology)  2. Usual pastoral care class | Adolescents (16.36); General population | 267 (17.23); ITT | School, Australia | NA | Moderate |  |
| Chong, Mak, Leung, Lam & Loke (2019) | ACT; 4 weekly sessions (120); Parent-only; Group sessions | 1. ACT (plus asthma education)  2. Asthma education | Children (6.80); Asthma | 168 (4.17); ITT | Clinical PH, China | 6 (3.57) | High |  |
| Ebrahiminejad, Poursharifi, Roodsari, Zeinodini & Noorbakhsh (2016) | MBCT; 8 weekly sessions (120); Child-only; Group sessions | 1. MBCT  2. No intervention/waitlist | Adolescents (14.95); Social anxiety | 30 (16.67); Completer | School, Iran | NA | Low |  |
| Esmaeilian, Dehghani, Dehghani & Lee (2018)** | MBCT; 12 weekly sessions (90); Child-only; Group sessions | 1. MBCT  2. No intervention/waitlist | Children and adolescents (12.13); Parental divorce | 83 (9.64); ITT | School, Iran | 2 (14.46) | High |  |
| Fang & Ding (2020) | ACT; 10 sessions over 5 weeks (60); Child-only; Group Sessions | 1. ACT  2. Control – school education course | Children and adolescents (13.23); Poverty-stricken area | 35 (5.41); Completer | School, China | NA | Moderate | |
|  |  |  |  |  |  |  |  |  |
| Faraji, Talepasand & Boogar (2019) | MBCT; 12 weekly sessions (90); Child-only; Group sessions | 1. MBCT  2. No intervention/waitlist | Children (NR); Bullying behaviour | 20 (NR); NR | School, Iran | NA | Low |  |
| Fatemi, Shafiabadi, Khalatbari & Farhangi (2021) | ACT; 12 weekly sessions (90); Child-only; NR | 1. ACT  2. Reality therapy based on Choice Theory  3. No intervention | Adolescents (16.6); low communication skills | 45 (NR); NR | School, Iran | NR | Low | |
|  |  |  |  |  |  |  |  |  |
| Hancock et al. (2018); Swain, Hancock, Dixon & Bowman. (2015) | ACT; 10 weekly sessions (90); Joint parent-child involvement; Group sessions | 1. ACT  2. CBT  3. No intervention/waitlist | Children and adolescents (11.00); Anxiety disorder | 193 (18.13); ITT (partial) | Clinical MH, Australia | 3 (18.65) | High |  |
| Hayes, Boyd & Sewell (2011) ** | ACT; 21 hours of sessions average; Child-only; Individual sessions | 1. ACT  2. TAU (psychotherapy/CBT) | Adolescents (14.90); Depression | 38 (21.05); ITT variant | Clinical MH, Australia | 3 (68.42) | Moderate |  |
| Kallesoe, Schroder, Jensen, Wicksell & Rask (2021)** | ACT; 27hrs over 3-months + one follow up meeting; Child-only; Group sessions | 1. ACT  2. Enhanced Usual Care | Adolescents; (17.9); Multiple functional somatic syndromes | 91 (10.99); Completer | Hospital, Denmark | 7 (6.59) | High | |
|  |  |  |  |  |  |  |  |  |
| Karekla, Nikolaou & Merwin (2022)** | ACT; 6 sessions (30) each session minimum 3 days apart; Child-only; Individual Sessions | 1. ACT (AcceptMe)  2. Waitlist | Adolescents (15.3); Female, eating disorder | 92 (36.96); ITT | School/online, Cyprus | 1 (42.39) | Moderate | |
|  |  |  |  |  |  |  |  |  |
| Kashefinishabouri, Saadi, Pasha, Heidari & Makvandi (2021) | MBCT; 8 weekly sessions (120); Child-only; Group sessions | 1. MBCT  2. Emotion Regulation Training  3. No intervention | Adolescents; (15.53); Risk of addiction | 45 (NR); NR | Foster care centers, Iran | 2 (NR) | Moderate | |
|  |  |  |  |  |  |  |  |  |
| Lee et al. (2020)** | ACT; 10 sessions (50); Joint parent-child; Individual sessions | 1. ACT  2. Waitlist | Adolescent (NR); Trichotillomania | 14 (21.43); ITT | Research clinic, USA | NA | Moderate | |
|  |  |  |  |  |  |  |  |  |
| Livheim et al. (2015) – Study A** | ACT; 8 weekly sessions; Child-only; Group sessions | 1. ACT  2. Usual school care (support from school counsellor) | Adolescents (14.6); Depression | 66 (22.73); ITT | School, Australia | NA | Moderate |  |
| Livheim et al. (2015) – Study B | ACT; 8 sessions over 6 weeks (90); Child-only; Group sessions | 1. ACT  2. Usual school care (sessions with school nurse) | Adolescents (NR); Stress | 32 (21.88); ITT | School, Sweden | NA | Low |  |
| Moazzezi, Moghanloo, Moghanloo & Pishvaei (2015) | ACT; 10 weekly sessions (90); Child-only; Individual sessions | 1. ACT  2. No intervention/waitlist | Children and adolescents (10.58); Diabetes Mellitus | 40 (20); NR | Clinical PH, Iran | NA | Low |  |
| Moghanloo, Moghanloo & Moazzezi (2015) | ACT; 10 weekly sessions (90); Child-only; Group sessions | 1. ACT  2. No intervention/waitlist | Children and adolescents (10.47); Diabetes Mellitus | 40 (15); NR | Clinical PH, Iran | NA | Low |  |
| Pahnke, Lundgren, Hursti & Hirvikoski (2014)* | ACT; 12 sessions over 6 weeks (40), Child-only; Group sessions | 1. ACT  2. No intervention/waitlist | Adolescents (16.50); Autism | 28 (0); NA | School, Sweden | 2 (0) | Moderate |  |
| Puolakanaho et al. (2019); Kiuru et al. (2021); Lappalainen et al. (2021) | ACT; 5 week online intervention (average total web time 84mins) with additional virtual/face-to-face support; Child-only; Individual sessions | 1. iACT online plus face-to-face  2. iACT online only  3. Usual school support  (Groups 1+2 combined for this review) | Adolescents (15.27); General population | 249 (4.02); ITT | School/online, Finland | 6 (10.04) | High |  |
| Quchani, Arbabi & Smaeili (2021)** | ACT; 8 weekly sessions over 2 months (60); Parents-only; Group sessions | 1. ACT  2. Lynn Clark Intervention Program  3. Regular medical care | Children (7.38); Divorced single parents | 66 (0); NA | Counselling centres, Iran | NA | Moderate |  |
|  |  |  |  |  |  |  |  |  |
| Raes, Griffith, Van der Gucht & Williams (2014)* | MBCT; 8 weekly sessions (100); Child-only; Group sessions | 1. MBCT (plus MBSR)  2. No intervention/waitlist | Adolescents (15.40); Depression | 408 (9.31); Partial ITT | School, Belgium | 6 (15.44) | Low |  |
| Razaeisharif, Cheraghian & Naeim (2021) | ACT; 8 weekly sessions (90); Child-only; Group sessions | 1. ACT  2. No intervention | Adolescents (NR); Body image disorder | 280 (NR); Completer | School, Iran | NA | Low |  |
| Reddy et al. (2012) | CFT; 2 sessions per week for 6 weeks (60); Child-only; Group sessions | 1. Cognitive-based Compassion Training  2. No intervention/waitlist | Adolescents (14.70); Looked after children | 70 (NR); NR | Foster care system, USA | NA | Low |  |
| Sairanen, Lappalainen, Lappalainen &Hiltunen (2022) | ACT; 10 weekly sessions (NR); Parent-only; Individual (plus group discussion forum) | 1. ACT-based online intervention  2. No intervention/waitlist | Children and adolescents (10.3); Diabetes or functional disabilities | 66 (NR); NR | Paediatric Clinic, Sweden | 4 (NR) | Moderate |  |
|  |  |  |  |  |  |  |  |  |
| Shabani et al. (2019) ** | ACT; 10 weekly sessions (60); Joint parent-child involvement; Group sessions | 1. ACT + SSRI  2. CBT + SSRI  3. SSRI | Adolescents (14.96); Obsessive compulsive disorder | 69 (7.25); ITT | Clinical MH, Iran | 3 (20.29) | High |  |
| Shetty, Kongasseri & Rai (2020)** | MBCT-C; 12 weekly sessions (NR); Joint parent-child; Group Sessions | 1. MBCT-C  2. Group therapy including cognitive behavioural principles | Children (9); Anxiety | 52 (0); NA | School, India | NA | High | |
|  |  |  |  |  |  |  |  |  |
| Shokri, Kazemi, Narimani, Taklavi (2022)*, ** | MBCT; 8 weekly sessions (120); Parent-only; Group sessions | 1. MBCT  2. James Gross’s cognitive emotion regulation protocol  3. No intervention | Children (NR); Externalising problems and self-efficacy in aggressive children | 60 (NR); NR | School, Iran | NA | Moderate |  |
| Simon, Driessen, Lambert & Muris (2019) | ACT; 1 session (30); Child-only; Individual sessions | 1. ACT (cognitive defusion)  2. CBT (cognitive restructuring) | Children (9.33); Fear of the dark | 43 (0); NA | Community, Belgium | NA | Low |  |
| Simons, Schneider & Herpertz-Dahlmann (2006) | MCT; up to 20 weekly sessions; Joint parent-child involvement; Individual sessions | 1. MCT  2. Narrative Exposure and Response Prevention | Children and adolescents (13.96); obsessive compulsive disorder | 11 (9.09); NR | Clinical MH, Germany | 24 (18.18) | Low |  |
| Syeda, Andrews (2021)** | MBCT-C; 12 weekly sessions (90); Parent involvement; Group sessions | 1. MCBT-C  2. No intervention/waitlist | Children (10.20); Anxiety | 25 (24); Completer | Community, Canada | 1 (24) | Moderate |  |
|  |  |  |  |  |  |  |  |  |
| Sveen, Andersson, Buhrman, Sjoberg & Willebrand (2017) | ACT; 6 weekly internet modules; Parent-only; Individual sessions | 1. ACT (and CBT components)  2. No intervention/waitlist | Children and adolescents (5.83); Burns | 104 (61.54); Partial ITT | Clinical PH, Sweden | 12 (58.65) | Low |  |
| Talaeizadeh (2020) | ACT; 8 sessions, twice a week (NR); Child-only: Individual sessions | 1. ACT  2. CBT  3. No intervention | Adolescents 13-17 (NR); Poor mental wellbeing | 45 (0); NA | School, Iran | NA | Low |  |
|  |  |  |  |  |  |  |  |  |
| Twohig et al. (2021) | ACT; 10 sessions (50); Joint parent-child; Individual sessions | 1. ACT (combined with habit reversal training)  2. No intervention/waitlist | Adolescents (13.9); Trichotillomania | 28 (7.14); ITT | Online, USA | NA | Moderate |  |
|  |  |  |  |  |  |  |  |  |
| Usubini et al. (2022) | ACT; 3 weekly sessions (60) + TAU; Child-only; Individual sessions | 1. ACT + TAU  2. TAU | Adolescents (15.55); obesity | 49 (30.61%); Completer | Hospital, Italy | NA | Moderate |  |
|  |  |  |  |  |  |  |  |  |
| Van der Gucht et al. (2017)* | ACT; 4 weekly sessions (120); Child-only; Group sessions | 1. ACT  2. Usual school curriculum | Adolescents (17.00); Non-clinical/mental health promotion | 586 (8.36); NR | School, Belgium | 12 (34.98) | High |  |
| Veysi, Rostami, Zangooi & Beldachi (2015) | MBCT; 12 weekly sessions (120); Child-only; Group sessions | 1. MBCT  2. No intervention/waitlist | Adolescents (13.85); Learning difficulties | 40 (NR); NR | School, Iran | NA | Low |  |
| White, Lubans & Eather (2022)* | ACT; 5 hours per fortnight for 20-weeks; Child-only; Group sessions | 1. Act + Self Determination Theory  2. Alternative elective school course | Adolescents; (14); School children | 90 (1.1); Completer | School, Australia | NA | Moderate |  |
|  |  |  |  |  |  |  |  |  |
| Whittingham, Sanders, McKinlay & Boyd (2014; 2016; 2019) | ACT; 2 sessions (120); Parent-only; Group sessions | 1. ACT + SSTP  2. SSTP  3. No intervention/waitlist | Children (5.30); Cerebral palsy | 80 (28.75); ITT | Clinical PH, Australia | 6 (66.25) | Moderate |  |
| Wicksell, Melin, Lekander, & Olsson (2009); Wicksell, Olsson & Hayes (2011) | ACT; 10 weekly child sessions (60) plus 1-2 parent sessions (90); Joint parent-child involvement; Individual sessions | 1. ACT  2. TAU (MDT approach + pain medication) | Children and adolescents (14.80); Pain | 32 (9.38); ITT | Clinical PH, Sweden | 6.5 (25) | Moderate |  |
| Wright, Roberts & Proeve (2019) | MBCT; 10 weekly sessions plus 2 meeting for parents and weekly emails encouraging practice; Joint parent-child involvement; Group sessions | 1. MBCT  2. CBT | Children and adolescents (10.60); Internalising difficulties | 89 (4.49); ITT | School, Australia | NA | High |  |
| Xu, Shen & Wang (2021) | ACT; 8 x 3 weekly sessions (40-60); Child-only; Group sessions | 1. ACT combined with aerobic exercise  2. No intervention | Child and Adolescent; (13.99); distress in response to COVID-19pandemic | 90 (7.78); NR | Community, China | NA | Low |  |
|  |  |  |  |  |  |  |  |  |
| Xu, Zhu & Liu (2019) | MBCT; 8 weeks with a minimum of 6 minutes of training per day; Child-only; Individual sessions | 1. MBCT  2. No intervention/waitlist | Adolescents (16.75); Mild depression | 36 (11.11); Completer | School, China | NA | Moderate |  |

ACT=Acceptance and Commitment Therapy; CFT=Compassion Focused Therapy; MBCT=Mindfulness-Based Cognitive Therapy; MCT=Metacognitive Therapy; SSTP=Stepping Stones Triple P; TAU=Treatment as Usual; CBT=Cognitive Behavioural Therapy; MBSR=Mindfulness-Based Stress Reduction; SSRI=Selective serotonin reuptake inhibitor; M=mean; NR=not reported in study; N=number of participants; ITT=intention-to-treat; NA=not applicable/measured in study

†Children=0-12 years; Adolescents=13-18 years

*cluster randomised studies

**studies rated highly for intervention quality

***see Supplementary Material 6 for studies references not cited in the main manuscript

Supplementary Material 2: Quality Assessment Process

The Cochrane risk-of-bias tool for randomised trials (Version 2) (Sterne et al., 2019) or for cluster-randomised designs (Eldridge et al., 2016), which categorise studies as “low risk of bias”, having “some concerns” or as “high risk of bias”, were supplemented with the following appraisal items from the NICE quality appraisal checklist for quantitative intervention studies (NICE, 2012), given they capture additional information essential for consideration in meta-analyses:

1. Is the source population or source area well described?
2. Is the eligible population or area representative of the source population or area?
3. Do the selected participants or areas represent the eligible population or area?
4. Were interventions (and comparisons) well described and appropriate?

*(Assessors were specifically asked to consider whether the intervention was: i) specific to third wave CBT or combined with other interventions not relevant to the review; ii) manualized and comprehensive (e.g. covering all relevant content or methods) or non-standardised and incomplete (e.g. ACT interventions focused on defusion only); and ii) carefully adapted to suit participants’ developmental level or lacking evidence of this)*

1. Was the study sufficiently powered to detect an intervention effect (if one exists)?

For each item from the NICE checklist, five categorical ratings were possible:

++ Indicates that for that particular aspect of study design, the study has been designed or conducted in such a way as to minimise the risk of bias.

+ Indicates that either the answer to the checklist question is not clear from the way the study is reported, or that the study may not have addressed all potential sources of bias for that particular aspect of study design.

− Should be reserved for those aspects of the study design in which significant sources of bias may persist.

Not reported (NR) Should be reserved for those aspects in which the study under review fails to report how they have (or might have) been considered.

Not applicable (NA) Should be reserved for those study design aspects that are not applicable given the study design under review (for example, allocation concealment would not be applicable for case control studies).

The researchers assigned an overall NICE category rating (++/+/-, using the same descriptions above) based on the five individual ratings. It was decided a priori that additional weight would be given to the item capturing intervention specificity and quality, given suggestions that third wave CBT is poorly researched and/or applied within child and adolescent populations, and the need to consider the impact of these factors in a review and meta-analysis aiming to determine effectiveness. For example, it has been argued that, in many instances, third wave CBT designed for adults has been “imported” to child populations without careful consideration of development (Hayes & Ciarrochi, 2015). Moreover, many combine third wave CBT with other approaches in research (e.g. Sveen, Andersson, Buhrman, Sjoberg & Willebrand 2017).

The overall categorical ratings from both the Cochrane tool and NICE appraisal checklist were merged, and studies were given a final category rating of high, moderate, or low quality:

- High: All or most of the criteria, across both the NICE and Cochrane checklists, scored well, where they have not met criteria the conclusions are very unlikely to alter or not meeting criteria were unavoidable
- Moderate: Some of the criteria across both the NICE and Cochrane checklists have scored well, and where they have not, or haven’t been adequately described, the conclusions are unlikely to alter.
- Low: Few or no checklist criteria have been fulfilled across either the NICE or Cochrane checklists, and the conclusions are likely or very likely to alter.

*(Criteria were adapted from the NICE quality appraisal checklist for quantitative intervention studies).*

Details of study quality rating scores across individual criteria are available on request.

Supplementary Material 3: Measures by Outcome Category

| Category | Example Measures (list not exhaustive) |
| --- | --- |
| Emotional Symptoms and Internalising Problems | Perceived Stress Scale; Child Stress Reaction Checklist – Short Form; Childhood Trauma Questionnaire; Children’s Yale-Brown Obsessive Compulsive Scale; Social Anxiety Scale; State-Trait Anxiety Inventory; Children’s Depression Inventory; Reynold’s Adolescent Depression Scale-2; Affective Control Scale; Difficulties with Emotion Regulation Scale; Strengths and Difficulties Questionnaire; Revised Child Anxiety and Depression Scale; Body Shape Questionnaire; Spence Children’s Anxiety Scale; Rumination Scale |
| Behavioural Difficulties and Externalising Problems | Child Behaviour Checklist; Eyberg Child Behaviour Inventory; Attention Control Scale; Inventory of Interpersonal Problems; State-Trait Anger Expression Inventory-2; Inventory of Callous and Unemotional Traits for Youth; Difficulties with Emotion Regulation Scale; Strengths and Difficulties Questionnaire; Relational Aggression Scale |
| Interference from Difficulties | Pain Interference Scale; Pain and Impairment Relationship Scale; Children’s Anxiety Life Interference Scale; Child and Youth Resilience Measure; Avoidance and Fusion Questionnaire Youth; Illness Related Behaviour Scales |
| Third Wave Processes | Meta-Cognitions Questionnaire; Child and Adolescent Mindfulness Measure; Mindful Attention Awareness Scale; Acceptance and Action Questionnaire; Avoidance and Fusion Questionnaire Youth; Self-Compassion Scale |
| Wellbeing and Flourishing | Valued Living Questionnaire; Flourishing Scale; Children’s Hope Scale; Meaning in Life Questionnaire; Social Connectedness Scale; Rosenberg Self-Esteem Scale; Meaning in Life Questionnaire; Positive Psychological Capital Questionnaire; Valuing Questionnaire; Warwick Edinburgh Mental Wellbeing Scale; Psychological Flourishing Scale; Oxford Happiness Questionnaire |
| Quality of Life | Satisfaction with Life Scale; Student’s Life Satisfaction Scale; Affect Balance Scale; Pediatric Quality of Life, Enjoyment and Satisfaction Questionnaire; Child Health Questionnaire; KINDL Quality of Life |
| Physical Health and Pain | Functional Disability Inventory; Pain Intensity Rating; Pain Coping Questionnaire; Short Form (36) Health Survey; Special Health Self-Efficacy Scale; Hospital Visits; BMI; Number of Days with Symptoms; Illness Severity Symptom Checklist Revised |

NB: Some measures were relevant across multiple categories (e.g. Strengths and Difficulties Questionnaire). Where subscales were presented alongside total score and not all subscales were relevant to an outcome category, only those relevant subscale(s) were inputted into analyses. Where multiple subscales or measures were relevant to an outcome category, an average effect size was calculated for these and inputted into analyses.

There were eight child outcome measures not extracted and included in analyses across studies meeting inclusion criteria, given they did not fit into any of the pre-determined outcome categories above. These are listed below for transparency:

1. Parent-Child Relationship Scale, measuring perceived quality of the relationship between children and parents. Reported in Azizi, Sepehri & Demehri (2021).
2. Interpersonal Conflict Resolution Assessment, a performance test of interpersonal problem solving skills. Reported in Bernal-Manrique, Garcia-Martin & Ruiz (2020).
3. Utrecht Work Engagement Scale-Student, assessing school engagement. Reported in Fang & Ding (2020).
4. Communication Skills Test-Revised capturing communication skills (all subscales except for emotion regulation which was extracted). Reported in Fatemi, et al., (2021).
5. Preparedness for Career Setbacks, measuring inoculation against setbacks. Reported in Kiuru et al., (2021).
6. Youth Schema Questionnaire-Short Form, capturing early maladaptive schemas. Reported in Veysi et al., (2015).
7. Positive Affect Control Scale, measuring lack of regulation when experiencing positive emotions. Reported in Veysi et al., (2015).
8. CNS Vital Signs (Shifting Attention and Continuous Performance), measuring cognitive ability. Reported in Wright, Roberts & Proeve (2019).

Supplementary Material 4: Further Sensitivity Analyses

Main effects for primary outcomes with cluster randomised trials excluded

|  |  |  |  |  |  |  |  |
| --- | --- | --- | --- | --- | --- | --- | --- |

|  |  |  | k | Hedge’s *g*† | 95% CI | Significance | Heterogeneity *I^2^* (*Q with p-val*) |
| --- | --- | --- | --- | --- | --- | --- | --- |
| Emotional symptoms and internalising problems | |  | **38** | **-0.76** | **-1.11 to -0.41** | **<.001** | 93% (405.38, <.001) |
| Behavioural difficulties and externalising problems | | | **19** | **-0.73** | **-1.20 to -0.26** | **.003** | 92% (150.61, <.001) |
| Interference from difficulties | | | **20** | **-0.48** | **-0.91 to -0.05** | **.029** | 92% (251.88, <.001) |
| Third wave processes | | | **20** | **0.44** | **0.19 to 0.68** | **<.001** | 77% (71.49, <.001) |
| Wellbeing and flourishing | | | **17** | **0.91** | **0.41 to 1.41** | **<.001** | 92% (130.20, <.001) |
| Quality of life | | | 11 | 0.68 | -0.10 to 1.45 | .086 | 96% (78.38, <.001) |
| Physical health and pain | | | **7** | **0.92** | **0.02 to 1.81** | **.045** | 95% (128.33, <.001) |

Note: k=number of studies; CI=confidence interval; *I2=*percentage heterogeneity

†For emotional symptoms/internalising problems, behavioural difficulties/externalising problems, and interference from difficulties, a negative effect size favours the intervention group whilst a positive effect size favours the control comparison. For the remaining outcomes (third wave processes, wellbeing/flourishing, quality of life, and physical health/pain), a positive effect size favours the intervention group whilst a negative effect favours the control comparison.

Significant effects are denoted in bold.

Supplementary Material 5: Inspection of Publication Bias

i) Rank correlation tests of funnel plot asymmetry for outcomes at post-treatment

| Outcome | $\tau$ | Significance |
| --- | --- | --- |
| Emotional Symptoms and Internalising Problems | -.27 .010 | |
| Behavioural Difficulties and Externalising Problems | -.46 .002 | |
| Interference from Difficulties | -.10 .571 | |
| Third Wave Processes | .30 .054 | |
| Wellbeing and Flourishing | .58 <.001 | |
| Quality of Life | .39 .086 | |
| Physical Health and Pain | .28 .359 | |

Note: $\tau$=Kendall’s tau

ii) Funnel plots (random effects models) for primary outcome variables at post-treatment. Open circles (if any) show missing null studies estimated with the trim-and-fill method.

| 1. **Emotional Symptoms and Internalising Problems**   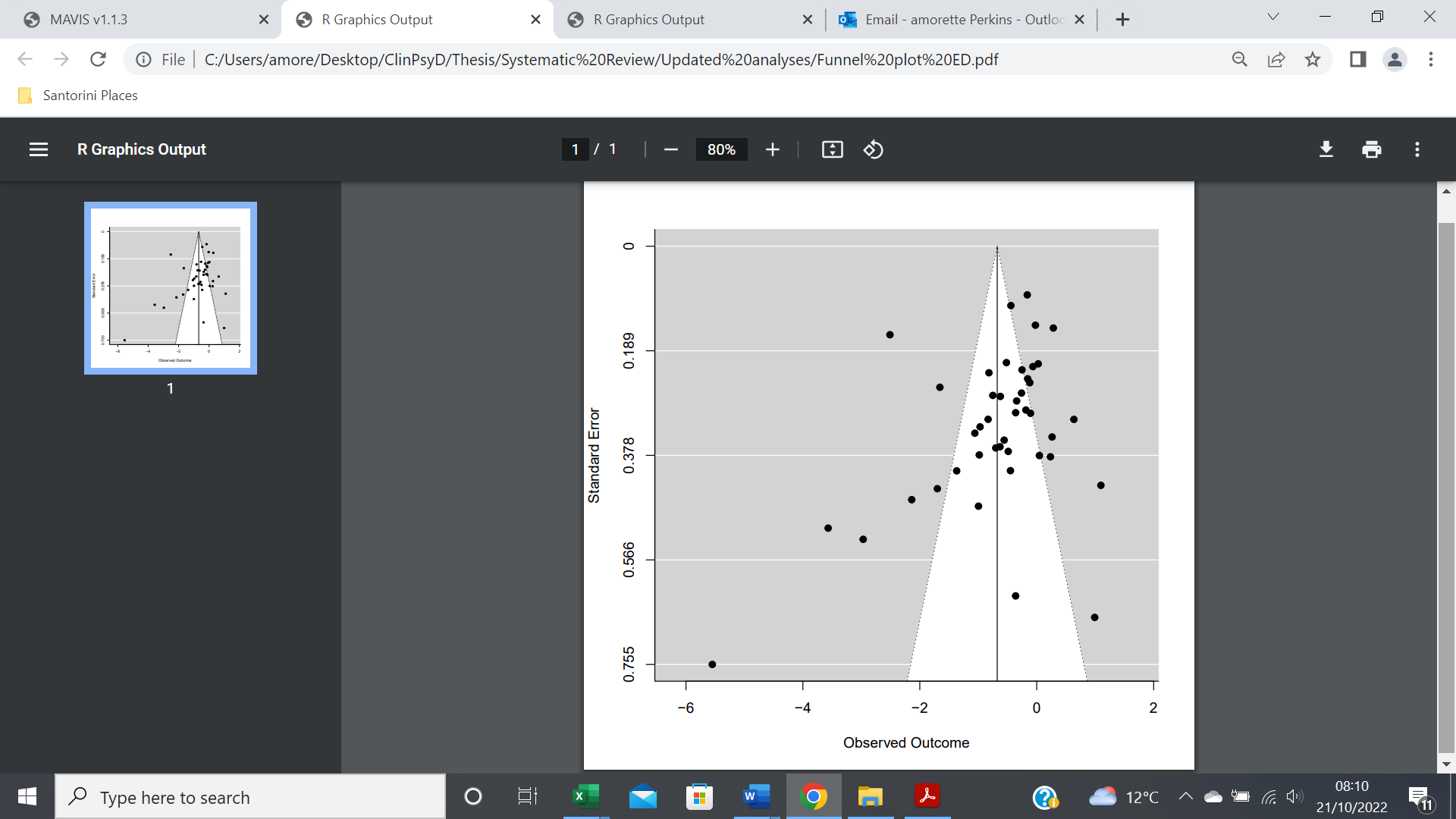 | 1. 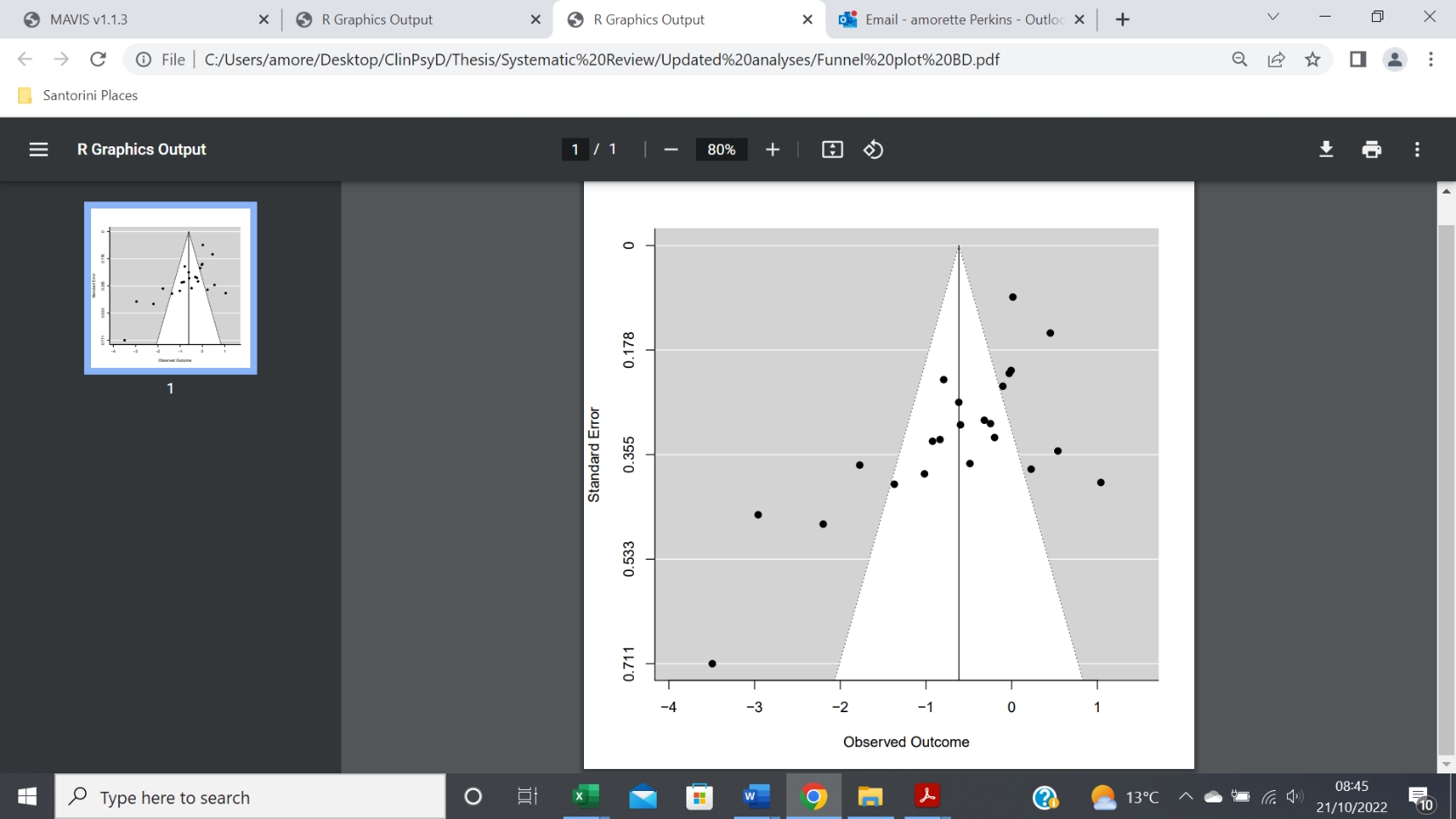**Behavioural Difficulties and Externalising Problems** |
| --- | --- |

| 1. **Interference from Difficulties**   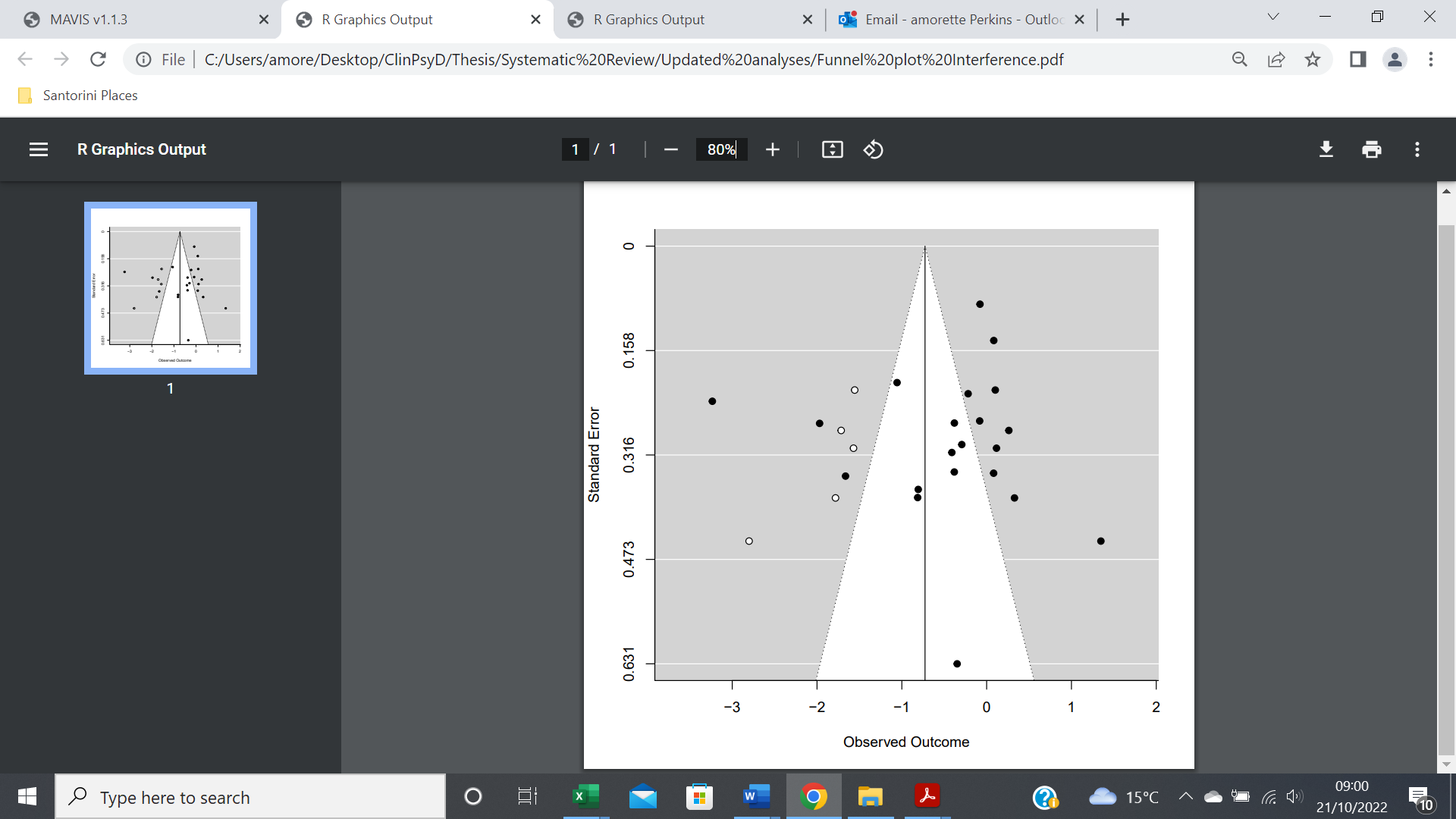 | 1. **Third Wave Processes**   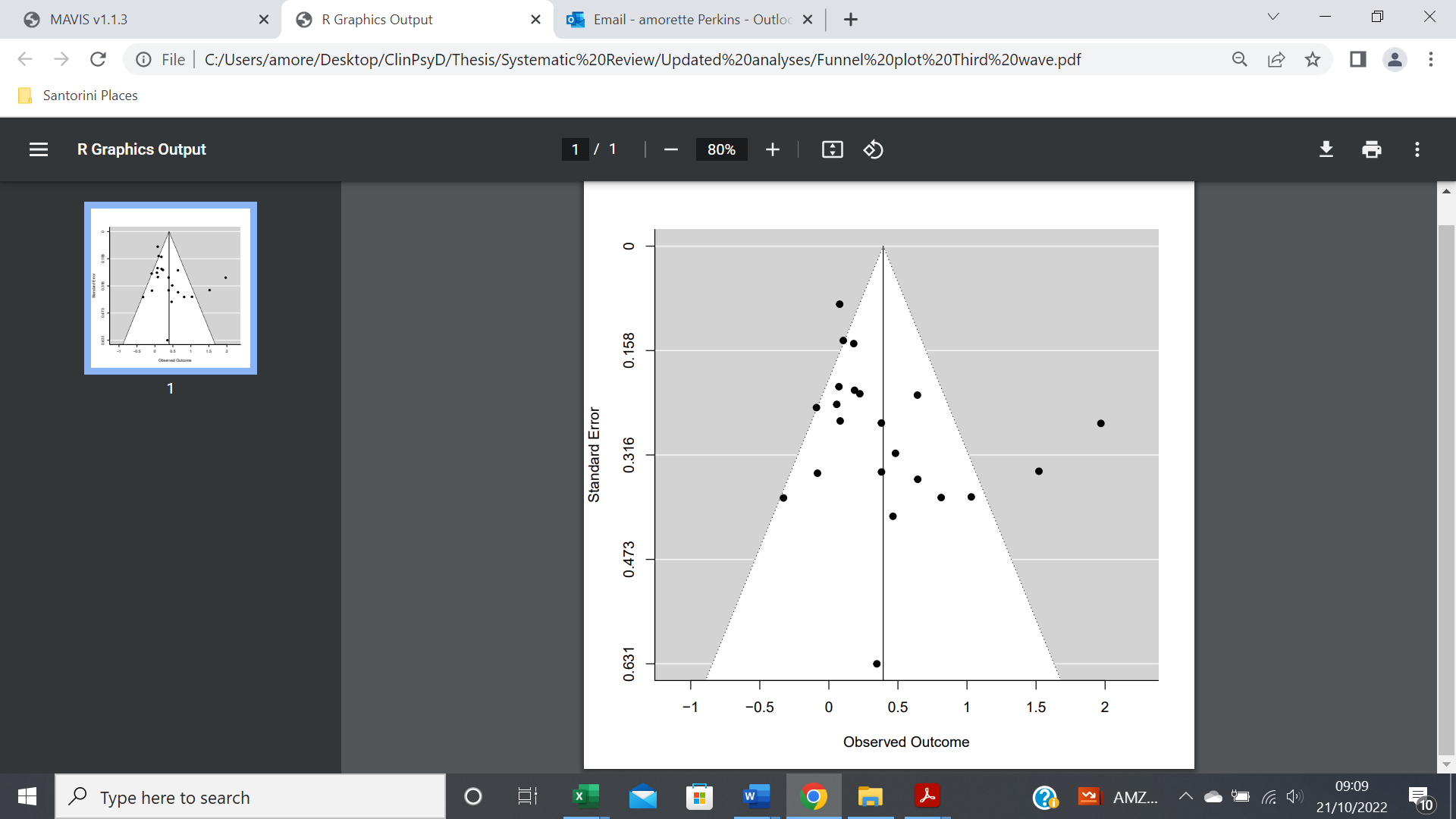 |
| --- | --- |
| 1. **Wellbeing and Flourishing**   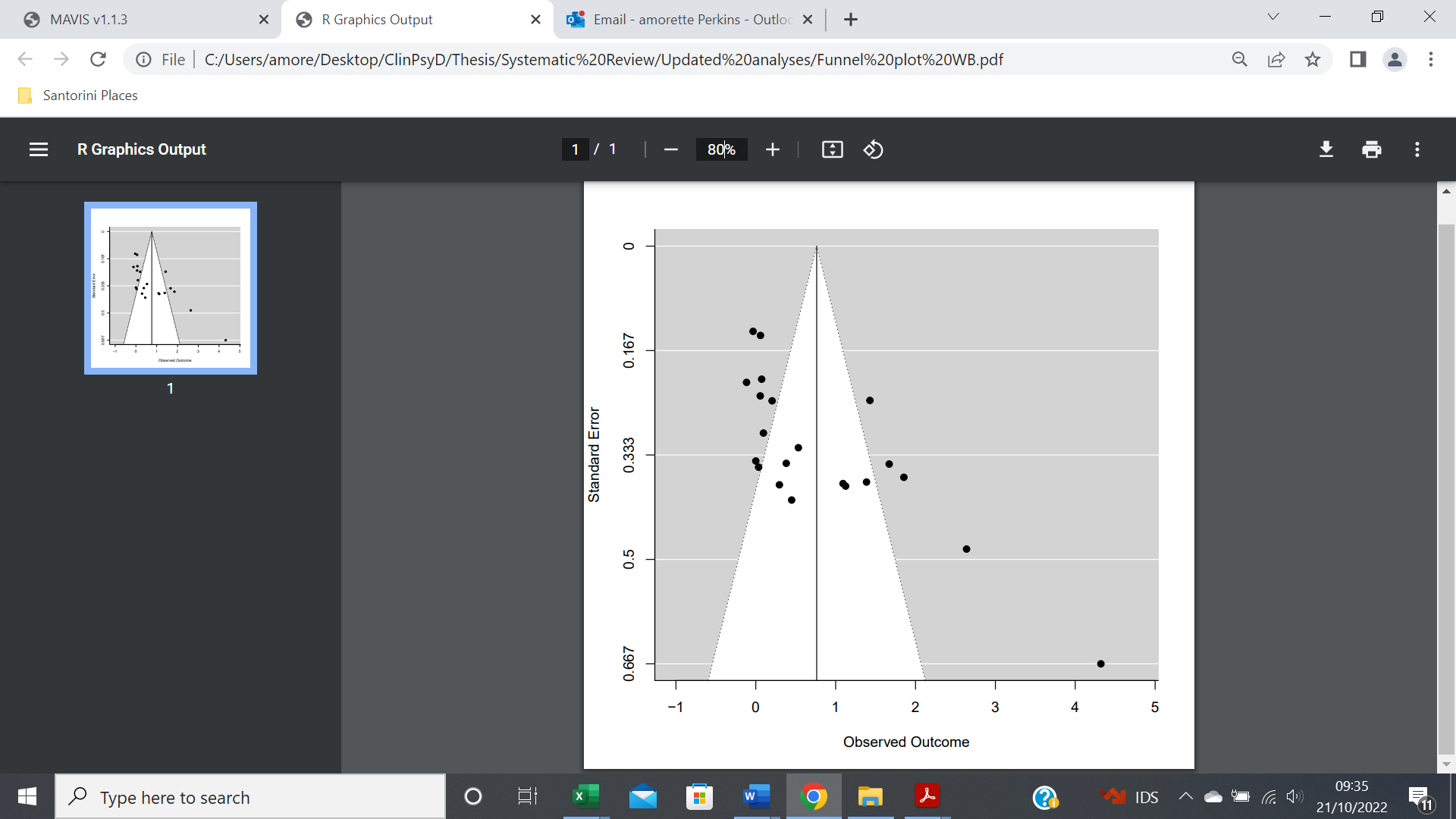 | 1. **Quality of Life**   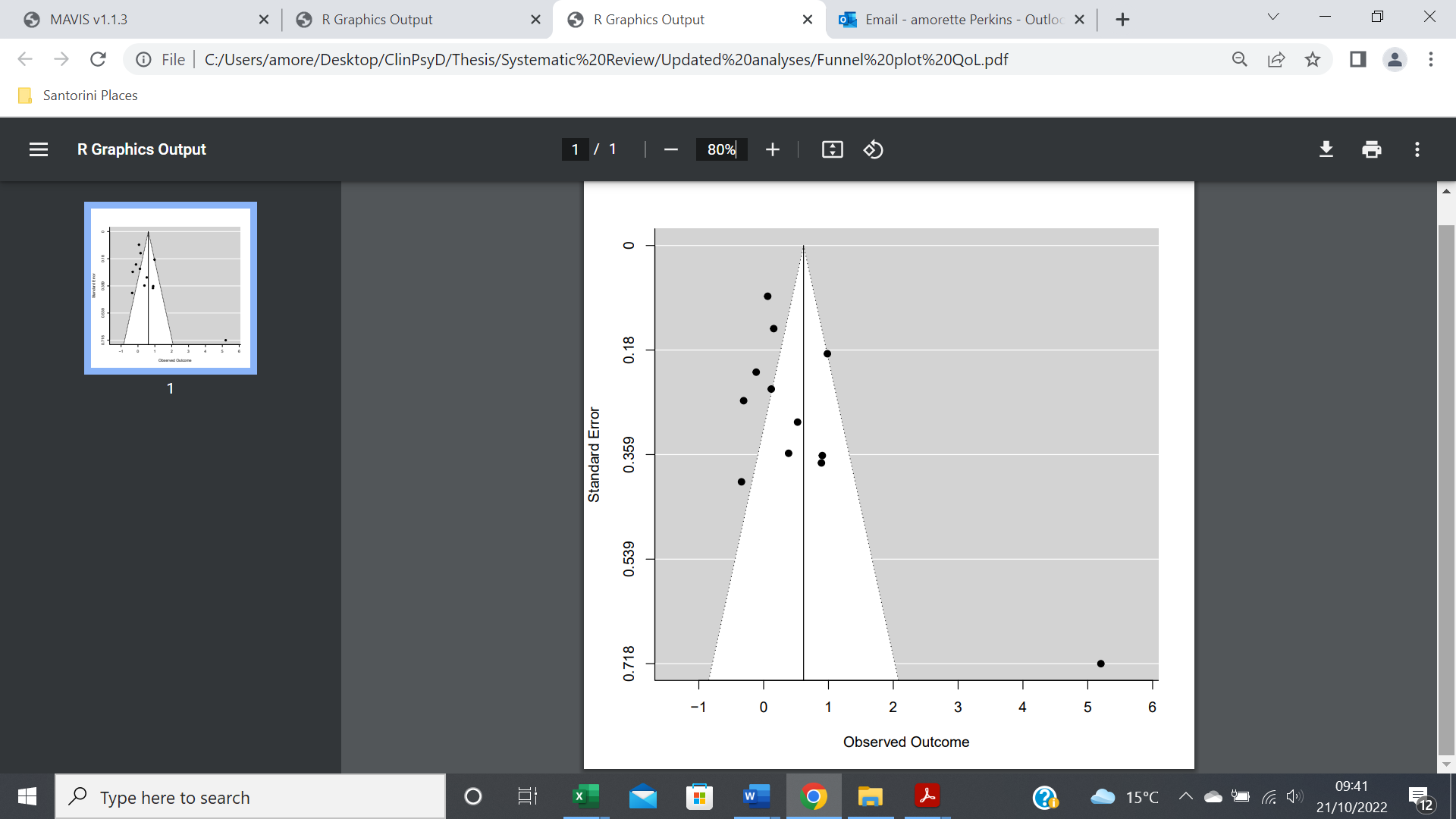 |
|  |  |

1. **Physical Health and Pain**


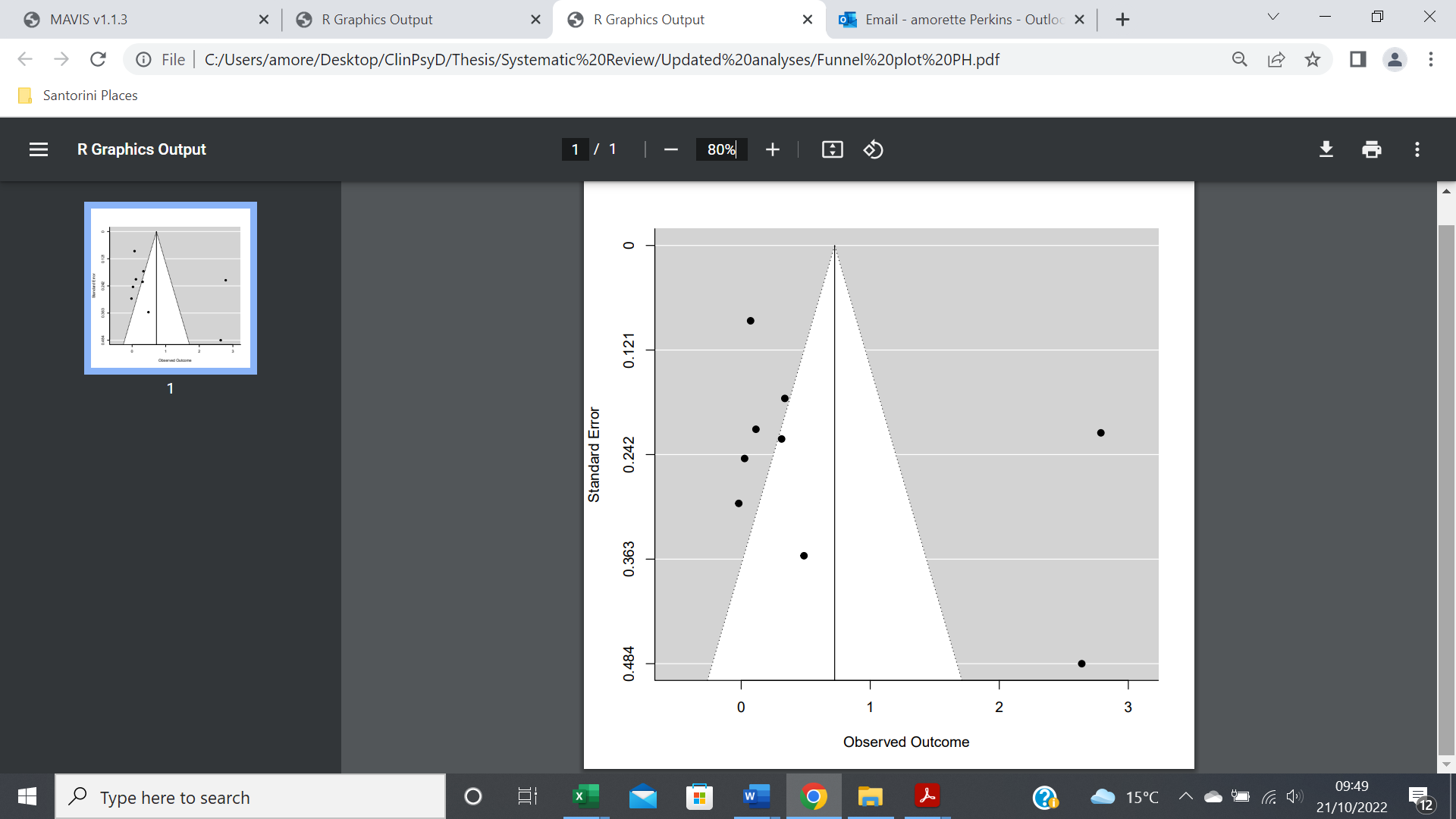


Supplementary Material 6: Additional References

(Those included in the meta-analysis/appendices but not cited in the main text)

Abedini, S., Habibi, M., Abedini, N., Achenbach, T. M. & Semple, R. J. (2021). A Randomized Clinical Trial of a Modified Mindfulness-Based Cognitive Therapy for Children Hospitalized with Cancer. *Mindfulness, 12*(1), 141-151. Doi: 10.1007/s12671-020-01506-3

Alampay, L. P., Tan, L. J. T., Tuliao, A. P., Baranek, P., Ofreneo, M. A., Lopez, G. D. … Guintu, V. (2019). A Pilot Randomised Controlled Trial of a Mindfulness Program for Filipino Children. *Mindfulness.* Doi: 10.1007/s12671-019-01124-8

Azadeh, S. M., Kazemi-Zahrani, H., & Besharat, M. A. (2016). Effectiveness of Acceptance and Commitment Therapy on Interpersonal Problems and Psychological Flexibility in Female High School Students with Social Anxiety Disorder. *Global Journal of Health Science, 8*(3), 131-138. Doi: 10.5539/gjhs.v8n3p131

Azizi, M., Sepehri, S., & Demehri, F. (2021). Effect of Acceptance and Commitment Therapy Combined with Compassion-Focused Therapy on Behavioral Problems and Mother-Child Interactions in Children with Hearing Impairment. *Auditory and Vestibular Research, 30*(4), 256-263. Doi: 10.18502/avr.v30i4.7473

Barandeh, N., Shafiabadi, A., & Farzad, V. (2017). A Comparison of the Effectiveness of Acceptance and Commitment Therapy (ACT) and Choice Theory on the Procrastination among Female Third-Grade High School Students in Shahriar County. *Journal of Fundamentals of Mental Health, 19,* 319-326

Bernal-Manrique, K. N., Garcia-Martin, M. B., & Ruiz, F. J. (2020). Effect of Acceptance and Commitment Therapy in Improving Interpersonal Skills in Adolescents: A Randomized Waitlist Control Trial. *Journal of Contextual Behavioral Science, 17*, 86-94. Doi: 10.1016/j.jcbs.2020.06.008

Bluth, K., Gaylord, S. A., Campo, R. A., Mullarkey, M. C., & Hobbs, L. (2016). Making Friends with Yourself: A Mixed Methods Pilot Study of a Mindful Self-Compassion Program for Adolescents. *Mindfulness, 7*(2), 479-492. Doi: 10.1007/s12671-015-0476-6

Brown, F. L., Whittingham, K., Boyd, R. N., McKinlay, L., & Sofronoff, K. (2014). Improving Child and Parenting Outcomes Following Paediatric Acquired Brain Injury: A Randomised Controlled Trial of Stepping Stones Triple P plus Acceptance and Commitment Therapy. *The Journal of Child Psychology and Psychiatry, 55*(10), 1172-1183. Doi: 10.1111/jcpp.12227

Chong, Y., Mak, Y., Leung, S., Lam, S., & Loke, A. Y. (2019). Acceptance and Commitment Therapy for Parental Management of Childhood Asthma: An RCT. *Pediatrics, 143*(2):e20181723. Doi: 10.1542/peds.2018-1723

Ebrahiminejad, S., Poursharifi, H., Roodsari, A. B., Zeinodini, Z., & Noorbakhsh, S. (2016). The Effectiveness of Mindfulness-Based Cognitive Therapy on Iranian Female Adolescents Suffering from Social Anxiety. *Iranian Red Crescent Medical Journal, 18*(11):e25116. Doi: 10.5812/ircmj.25116 Esmaeilian, N., Dehghani, M., Dehghani, Z., & Lee, J. (2018). Mindfulness-Based Cognitive Therapy Enhances Emotional Resiliency in Children with Divorced Parents. *Mindfulness, 9,* 1052-1062. Doi: 10.1007/s12671-017-0840-9

Fang, S., & Ding, D. (2020). The Efficacy of Group-Based Acceptance and Commitment Therapy on Psychological Capital and School Engagement: A Pilot Study Among Chinese Adolescents. *Journal of Contextual Behavioral Science, 16*, 134-143. Doi: 10.1016/j.jcbs.2020.04.005

Faraji, M., Talepasand, S., & Boogar, I. R. (2019). Effectiveness of Mindfulness-Based Cognitive Therapy for Child on Bullying Behaviours among Children. *International Archives of Health Sciences, 6*(1), 52-57. Doi: 10.4103/iahs.iahs_54_18

Hancock, K. M., Swain, J., Hainsworth, C. J., Dixon, A. L., Koo, S., & Munro, K. (2018). Acceptance and Commitment Therapy versus Cognitive Behaviour Therapy for Children with Anxiety: Outcomes of a Randomised Controlled Trial. *Journal of Clinical Child and Adolescent Psychology, 47*(2), 296-311. Doi: 10.1080/15374416.2015.1110822

Hayes, L., Boyd, C. P., & Sewell, J. (2011). Acceptance and Commitment Therapy for the Treatment of Adolescent Depression: A Pilot Study in a Psychiatric Outpatient Setting. *Mindfulness, 2,* 86-94. Doi: 10.1007/s12671-011-0046-5

Karekla, M., Nikolaou, P. & Merwin, R. M. (2022). Randomized Clinical Trial Evaluating AcceptME—A Digital Gamified Acceptance and Commitment Early Intervention Program for Individuals at High Risk for Eating Disorders. *Journal of Clinical Medicine, 11*(7), 1775. Doi: 10.3390/jcm11071775

Kiuru, N., Puolakanaho, A., Lappalainen, P., Keinonen, K., Mauno, S., Muotka, J. & Lappalainen, R. (2021). Effectiveness of Web-Based Acceptance and Commitment Therapy Program for Adolescent Career Preparation: A Randomized Controlled Trial. *Journal of Vocational Behaviour, 127*:103578. Doi: 10.1016/j.jvb.2021.103578

Lappalainen, R., Lappalainen, P., Puolakanaho, A., Hirvonen, R., Eklund, K. Ahonen, T., … & Kiuru, N. (2021). The Youth Compass -The Effectiveness of an Online Acceptance and Commitment Therapy Program to Promote Adolescent Mental Health: A Randomized Controlled Trial. *Journal of Contextual Behavioral Science, 20*, 1-12. Doi: 10.1016/j.jcbs.2021.01.007

Lee, E. B., Homan, K. J., Morrison, K. L., Ong, C. W., Levin, M. E., & Twohig, M. P. (2020). Acceptance and Commitment Therapy for Trichotillomania: A Randomized Controlled Trial of Adults and Adolescents. *Behavior Modification, 44*(1), 70-91. Doi: 10.1177/0145445518794366

Livheim, F., Hayes, L., Ghaderi, A., Magnusdottir, T., Hogfeldt, A., Rowse, J., … & Tengstrom, A. (2015). The Effectiveness of Acceptance and Commitment Therapy for Adolescent Mental Health: Swedish and Australiana Pilot Outcomes. *Journal of Child and Family Studies, 24*(4), 1016-1030. Doi: 10.1007/s10826-014-9912-9

Moazzezi, M., Moghanloo, V. A., Moghanloo, V. A. & Pishvaei, M. (2015). Impact of Acceptance and Commitment Therapy on Perceived Stress and Special Health Self-Efficacy in Seven to Fifteen-Year-Old Children with Diabetes Mellitus. *Iranian Journal of Psychiatry, 9*(2):e956. Doi: 10.17795/ijpbs956

Moghanloo, V. A., Moghanloo, R. A., & Moazzezi, M. (2015). Effectiveness of Acceptance and Commitment Therapy for Depression, Psychological Well-Being and Feeling of Guilt in 7-15 Years Old Diabetic Children. *Iranian Journal of Pediatrics, 25*(4):e2436. Doi: 10.5812/ijp.2436

Puolakanaho, A., Lappalainen, R., Lappalainen, P., Muotka, J. S., Hirvonen, R., Eklund, K. M., … & Kiuru, N. (2019). Reducing Stress and Enhancing Academic Buoyancy among Adolescents Using a Brief Web-Based Program Based on Acceptance and Commitment Therapy: A Randomised Controlled Trial. *Journal of Youth and Adolescence, 48,* 287-305. Doi: 10.1007/s10964-018-0973-8

Quchani, M., Arbabi, F., H., & Smaeili, N., S. (2021). A Comparison of the Effectiveness of Clark and ACT Parenting Training on Improving the Emotional-Behavioral Problems of the Child with Divorced Single Mothers. *Learning and Motivation,* 76:101759. Doi: 10.1016/j.lmot.2021.101759

Raes, F., Griffith, J. W., Van der Gucht, K., & Williams, J. M. G. (2014). School-Based Prevention and Reduction of Depression in Adolescents: A Cluster-Randomised Controlled Trial of a Mindfulness Group Program. *Mindfulness, 5,* 477-486. Doi: 10.1007/s12671-013-0202-1

Rezaeisharif, A., Cheraghian, H., & Mahdi, N. (2021). Effectiveness of Acceptance and Commitment Therapy on Reducing Body Image Disorders in Adolescent Girls. *Addictive Disorders & Their Treatments, 20*(4), 336-341. Doi: 10.1097/ADT.0000000000000265

Reddy, S. D., Negi, L. T., Dodson-Lavelle, B., Ozawa-de Silva, B., Pace, T. W. W., Cole, S. P., … & Craighead, L. W. (2012). Cognitive-Based Compassion Training: A Promising Prevention Strategy for At-Risk Adolescents. *Journal of Child and Family Studies, 22*(2), 219-230*.* Doi: 10.1007/s10826-012-9571-7

Sairanen, E., Lappalainen, R., Lappalainen, P., & Hiltunen, A. (2022). Effects of an Online Acceptance and Commitment Therapy Intervention on Children’s Quality of Life. *Journal of Child and Family Studies,* *31*, 1079-1093. Doi: 10.1007/s10826-022-02234-z

Shabani, M. J., Mohsenabadi, H., Omidi, A., Lee, E. B., Twohig, M. P., Ahmadvand, A., & Zanjani, Z. (2019). An Iranian Study of Group Acceptance and Commitment Therapy versus Group Cognitive Behavioural Therapy for Adolescents with Obsessive-Compulsive Disorder on an Optimal Dose of Selective Serotonin Reuptake Inhibitors. *Journal of Obsessive-Compulsive and Related Disorders, 22:*100440. Doi: 10.1016/j.jocrd.2019.04.003

Shetty, R., Kongasseri, S., & Rai, S. (2020). Efficacy of Mindfulness Based Cognitive Therapy on Children with Anxiety. *Journal of Cognitive Psychotherapy: An International Quarterly, 32*(4), 306-318. Doi: 10.1891/JCPY-D-20-00014

Simon, E., Driessen, S., Lambert, A., & Muris, P. (2019). Challenging Anxious Cognitions or Accepting them? Exploring the Efficacy of the Cognitive Elements of Cognitive Behaviour Therapy and Acceptance and Commitment Therapy in the Reduction of Children’s Fear of the Dark. *International Journal of Psychology.* Doi: 10.1002/ijop.12540

Simons, M., Schneider, S., & Herpertz-Dahlmann, B. (2006). Metacognitive Therapy versus Exposure and Response Prevention for Pediatric Obsessive-Compulsive Disorder. *Psychotherapy and Psychosomatics, 75,* 257-264. Doi: 10.1159/000092897

Shokri, A., Narimani, M., & Taklavi, S. (2020). Comparison of the Effectiveness of Mother’s Mindfulness-based Cognitive Therapy and Cognitive Emotion Regulation Training on Externalizing Disorder and Self-efficacy of Aggressive Children. *Practice in Clinical Psychology, 8*(2), 85-98. Doi: 10.32598/jpcp.8.2.677.1

Sveen, J., Andersson, G., Buhrman, B., Sjoberg, F., & Willebrand, M. (2017). Internet-Based Information and Support Program for Parents of Children with Burns: A Randomised Controlled Trial. *Burns, 43,* 583-591. Doi: 10.1016/j.burns.2016.08.039

Talaeizadeh, F. (2020). Comparison of Acceptance Commitment Therapy (ACT) and Cognitive Behavioral Therapy (CBT) in Reducing Depression Symptoms and Increasing Happiness of Iranian Adolescent Girl Students. *Journal of Intellectual Disability – Diagnosis and Treatment*, *8*, 16-24. Doi: 10.6000/2292-2598.2020.08.01.3

Usubini, A., G., Cattivelli, R., Radaelli, A., Bottacchi, M., Landi, G., Tossani, E., … & Sartorio, A. (2022). Preliminary Results from the ACTyourCHANGE in Teens Protocol: A Randomized Controlled Trial Evaluating Acceptance and Commitment Therapy for Adolescents with Obesity. *International Journal of Environmental Research and Public Health, 19*, 5635. Doi: 10.3390/ijerph19095635

Fatemi, V. A., Shafiabadi, A., Khalatbari, J., & Farhangi, A. (2021). Comparison of the Effectiveness of Reality Therapy based on Choice Theory and Acceptance and Commitment Therapy on Communication Skills of Female High School Students. *Razavi International Journal of Medicine, 9*(4): e1063. Doi: 10.30483/RIJM.2021.254219.1063

Van der Gucht, K., Griffith, J. W., Hellemans, R., Bockstaele, M., Pascal-Claes, F., & Raes, F. (2017). Acceptance and Commitment Therapy (ACT) for Adolescents: Outcomes of a Large-Sample, School-Based, Cluster-Randomised Controlled Trial. *Mindfulness, 8,* 408-416. Doi: 10.1007/s12671-016-0612-y

Veysi, N., Rostami, M., Zangooi, Z., & Beldachi, M. A. K. (2015). Maladaptive Schemas and Affective Control in Students with Learning Disability: Benefits of Mindfulness-Based Cognitive Therapy. *Iranian Rehabilitation Journal, 13*(3), 77-83.

Whittingham, K., Sanders, M., McKinlay, L., & Boyd, R. N. (2014). Interventions to Reduce Behavioural Problems in Children with Cerebral Palsy: An RCT. *Pediatrics, 133*(5), e1249. Doi: 10.1542/peds.2013-3620

Whittingham, K., Sanders, M., McKinlay, L., & Boyd, R. N. (2016). Parenting Intervention Combined with Acceptance and Commitment Therapy: A Trial with Families of Children with Cerebral Palsy. *Journal of Pediatric Psychology, 41*(5), 531-542. Doi: 10.1093/jpepsy/jsv118

Whittingham, K., Sanders, M., McKinlay, L., & Boyd, R. N. (2019). Parenting Intervention Combined with Acceptance and Commitment Therapy: Processes of Change. *Journal of Child and Family Studies, 28,* 1673-1680. Doi: 10.1007/s10826-019-01386-9

Wicksell, R. K., Olsson, G. L., & Hayes, S. C. (2011). Mediators of Change in Acceptance and Commitment Therapy for Pediatric Chronic Pain. *Pain, 152,* 2792-2801. Doi: 10.1016/j.pain.2011.09.003

Wright, K. M., Roberts, R., & Proeve, M. J. (2019). Mindfulness-Based Cognitive Therapy for Children (MBCT-C) for Prevention of Internalising Difficulties: A Small Randomised Controlled Trial with Australian Primary School Children. *Mindfulness, 10*(11), 2277-2293. Doi: 10.1007/s12671-019-01193-9

Xu, W., Shen, W., & Wang, S. (2021). Intervention of Adolescent’ Mental Health During the Outbreak of COVID-19 Using Aerobic Exercise Combined with Acceptance and Commitment Therapy. *Children and Youth Services Review, 124*(51), 105960. Doi: 10.1016/j.childyouth.2021.105960

Xu, X-P., Zhu, X-W., & Lui, Q-Q. (2019). Can Self-Training in Mindfulness-Based Cognitive Therapy alleviate Mild Depression among Chinese Adolescents. *Social Behaviour and Personality, 47*(4), e7944. Doi: 10.2224/sbp.7944
